# Chromosomal-Level Assembly of the Asian Seabass Genome Using Long Sequence Reads and Multi-layered Scaffolding

**DOI:** 10.1371/journal.pgen.1005954

**Published:** 2016-04-15

**Authors:** Shubha Vij, Heiner Kuhl, Inna S. Kuznetsova, Aleksey Komissarov, Andrey A. Yurchenko, Peter Van Heusden, Siddharth Singh, Natascha M. Thevasagayam, Sai Rama Sridatta Prakki, Kathiresan Purushothaman, Jolly M. Saju, Junhui Jiang, Stanley Kimbung Mbandi, Mario Jonas, Amy Hin Yan Tong, Sarah Mwangi, Doreen Lau, Si Yan Ngoh, Woei Chang Liew, Xueyan Shen, Lawrence S. Hon, James P. Drake, Matthew Boitano, Richard Hall, Chen-Shan Chin, Ramkumar Lachumanan, Jonas Korlach, Vladimir Trifonov, Marsel Kabilov, Alexey Tupikin, Darrell Green, Simon Moxon, Tyler Garvin, Fritz J. Sedlazeck, Gregory W. Vurture, Gopikrishna Gopalapillai, Vinaya Kumar Katneni, Tansyn H. Noble, Vinod Scaria, Sridhar Sivasubbu, Dean R. Jerry, Stephen J. O'Brien, Michael C. Schatz, Tamás Dalmay, Stephen W. Turner, Si Lok, Alan Christoffels, László Orbán

**Affiliations:** 1 Reproductive Genomics Group, Temasek Life Sciences Laboratory, Singapore; 2 Max Planck Institute for Molecular Genetics, Berlin, Germany; 3 Laboratory of Chromosome Structure and Function, Department of Cytology and Histology, Biological Faculty, Saint Petersburg State University, St. Petersburg, Russia; 4 Theodosius Dobzhansky Center for Genome Bioinformatics, Saint Petersburg State University, St. Petersburg, Russia; 5 South African MRC Bioinformatics Unit, South African National Bioinformatics Institute, University of the Western Cape, Bellville, South Africa; 6 Pacific Biosciences, Menlo Park, California, United States of America; 7 Donnelly Centre for Cellular and Biomolecular Research, University of Toronto, Toronto, Canada; 8 Institute of Molecular and Cellular Biology, Siberian Branch of the Russian Academy of Sciences, Novosibirsk, Russian Federation; 9 Genomics Core Facility, Institute of Chemical Biology and Fundamental Medicine, Siberian Branch of the Russian Academy of Sciences, Novosibirsk, Russia; 10 Norwich Medical School, University of East Anglia, Norwich Research Park, Norwich, United Kingdom; 11 The Genome Analysis Centre, Norwich, United Kingdom; 12 Simons Center for Quantitative Biology, Cold Spring Harbor Laboratory, One Bungtown Road, Cold Spring Harbor, New York, United States of America; 13 Department of Computer Science, Johns Hopkins University, Baltimore, Maryland, United States of America; 14 Nutrition, Genetics & Biotechnology Division, ICAR-Central Institute of Brackishwater Aquaculture, Tamil Nadu, India; 15 College of Marine and Environmental Sciences and Center for Sustainable Tropical Fisheries and Aquaculture, James Cook University, Townsville, Queensland, Australia; 16 CSIR-Institute of Genomics and Integrative Biology (CSIR-IGIB), New Delhi, India; 17 Oceanographic Center, Nova Southeastern University Ft. Lauderdale, Ft. Lauderdale, Florida, United States of America; 18 School of Biological Sciences, University of East Anglia, Norwich Research Park, Norwich, United Kingdom; 19 The Centre for Applied Genomics, The Hospital for Sick Children, Peter Gilgan Centre for Research and Learning, Toronto, Ontario, Canada; 20 Department of Animal Sciences and Animal Husbandry, Georgikon Faculty, University of Pannonia, Keszthely, Hungary; 21 Centre for Comparative Genomics, Murdoch University, Murdoch, Australia; MicroTrek Incorporated, UNITED STATES

## Abstract

We report here the ~670 Mb genome assembly of the Asian seabass *(Lates calcarifer)*, a tropical marine teleost. We used long-read sequencing augmented by transcriptomics, optical and genetic mapping along with shared synteny from closely related fish species to derive a chromosome-level assembly with a contig N50 size over 1 Mb and scaffold N50 size over 25 Mb that span ~90% of the genome. The population structure of *L*. *calcarifer* species complex was analyzed by re-sequencing 61 individuals representing various regions across the species’ native range. SNP analyses identified high levels of genetic diversity and confirmed earlier indications of a population stratification comprising three clades with signs of admixture apparent in the South-East Asian population. The quality of the Asian seabass genome assembly far exceeds that of any other fish species, and will serve as a new standard for fish genomics.

## Author Summary

We describe the genome assembly of Asian seabass *(Lates calcarifer)*, a marine teleost with aquaculture relevance. Though >500 eukaryotic genome sequences are available in public repositories, the majority are highly fragmented with incomplete assemblies, which explains why considerable effort and resources are often spent to improve their quality after publication. In our study, we employed long read sequencing combined with genetic and optical mapping, and syntenic information to produce a chromosomal level assembly. The largely continuous genome assembly will be useful for comparative genomics and offers an opportunity to look into regions less explored such as tandem repeats (the core component of centromeres and telomeres). In addition, population structure of the species was analysed based on low-coverage genome sequence information from 61 individuals representing diverse geographic locations stretching from North-Western India across South-East Asia and Australia to Papua New Guinea.

## Introduction

The Asian seabass (*Lates calcarifer*; Bloch, 1790) is a highly fecund, robust, tropical species; immensely popular as a food fish in the Asia-Pacific and beyond. The species, which is also known as barramundi (Australia), pla kapong (Thailand), ikan siakap (Malaysia), and 75 other local names, is of significant cultural and economic importance through most of the tropical Indo-West Pacific region, as an important fishery target and as a commercially farmed species [1] (FAO 2011).

An opportunistic predator with a wide geographic range (Persian Gulf, SE Asia, India, Northern Australia, Papua New Guinea and the Western Pacific), the Asian seabass is a catadromous, euryhaline teleost that belongs to the Family Latidae [2]. Perhaps the most fascinating aspect of the species’ biology is its sequential hermaphroditic nature, with individuals typically maturing as males and later transforming their sex to become female [3–5]. Similar to other sex changers, limited information is available on the genetic basis of this sex change process in seabass. Given *L*. *calcarifer*’s (*senso lato*) wide geographical range across several known biogeographical barriers, it is also not surprising that an increasing body of evidence suggests the existence of a ‘species complex’ in the Indo-Pacific, rather than a single species [6–10].

The size of Asian seabass genome was estimated to be 700 Mb [11]. The karyotype is represented by a diploid number of A chromosomes (2n = 24) and a variable number (2–10) of additional B chromosomes [12]. Given the economic importance of the species and the needs of the selection program targeting polygenic traits, we embarked on the genome project with the main aim of employing next generation sequencing (NGS) platforms to produce a high-quality draft genome assembly. Mindful of the limitations of short sequencing reads, we chose to assemble the genome using Pacific Biosciences’ (PacBio; Menlo Park, CA, USA) long reads from single molecule, real-time (SMRT) sequencing [13,14] representing ~90X coverage of the genome. Multiple approaches were used to validate the assembly, including mapping Illumina (San Diego, CA, USA) paired-end reads (80X coverage) and alignment of BAC end sequences (~11,000) to the assembled genome. The N50 of the long–read based assembly was more than 1 Mb and contained <4,000 contigs. The genome was scaffolded using the assembled transcriptome in conjunction with optical mapping, a genetic map and synteny from closely related fish species to obtain a chromosomal-level assembly covering ~90% of the assembled sequence with a scaffold N50 of >25 Mb. In addition, to gain a better understanding of the genetic diversity, we obtained genome sequence information at shallow coverage from 61 seabass individuals whose origin spanned the species’ native range. We anticipate that the genome will be an important resource not only for the species itself (e.g. development of genomic assays for establishing molecular aquaculture) but also its relatives and other teleosts in general as affirmed by the observed chromosomal collinearity between Asian seabass, European seabass *(Dicentrarchus labrax)* and three-spined stickleback (*Gasterosteus aculeatus*).

## Results

### Long-read sequencing and assembly yielded a high quality draft genome of *L*. *calcarifer*

A partially inbred F2 Asian seabass specimen from SE Asia [10] was selected for genome sequencing. Fluorescence Activated Cell Sorting of liver cells from adult seabasses yielded a genome size of 734 +/- 66 Mb, while k-mer frequency counting estimated the haploid genome size to be 593–648 Mb. The k-mer analysis also revealed a relatively high rate of heterozygosity (0.4%-0.5%) resulting in a characteristic “double peak” in the k-mer frequency distribution (S1 Fig).

The genome sequence data was generated by two rounds of SMRT sequencing [14], yielding ~30X and ~60X (~4.5 kb and ~8 kb average read length) respectively, of genome coverage. The genome was assembled into 3,917 contigs totaling to 668.5 Mb in size (primary genome assembly; v1). The contig N50 value was over 1 Mb and 50% of the genome was represented in only 154 contigs (Table 1, S2 Fig). Although the genome information was obtained from a heterozygous individual, a diploid unaware assembler (Celera, as part of HGAP [15]) was used for assembling the genome, therefore, it was not possible to phase the variation between the maternal and paternal chromosomes.

**Table 1:** Assembly and scaffolding statistics for the Asian seabass genome.

Primary Genome Assembly (v1)		
	Number of contigs	3,917
	Contig N50/count	1,066,117/139
	Max. contig size	18,910,200
	Total size	668,453,369
Scaffolded Genome Assembly (v2)		
	Number of scaffolds	3,807
	Scaffold N50/count	1,191,366/119
	Max. scaffold size	18,910,200
	Total size	668,464,831
Chromosome-Level Genome Assembly (v3)		
	Number of chromosomes	24
	Scaffold N50/count	25,848,596/11
	Max. scaffold size	30,776,907
	Total size	586,924,032

Earlier, transcriptome sequence data was obtained from multiple platforms and assembled into 267,616 contigs [16]. The scaffolded genome assembly (v2) was obtained using the transcriptome [17] and yielded 3,807 genomic contigs resulting in a ~10% improvement in N50 metrics.

Evaluation of the genome for completeness based on CEGMA (Core Eukaryotic Genes Mapping Approach) [18,19] identified 88.7% complete and 98.8% partial genes from the 248 core eukaryotic genes dataset. Two paired-end (PE) libraries (~500 and ~750 bp insert size), sequenced on the Illumina platform were used to obtain an 80x coverage of the seabass genome. More than 95% of the reads mapped to the genome assembly in the expected orientation and in concordance with the expected paired-end distance (S3 Fig).

Of the 11,159 BAC end sequences (BES) that aligned to the genome, 81.3% were in pairs on the same scaffold, of which 78.7% aligned in the proper orientation and expected separation distance (50–250 kb). BES, which aligned to different scaffolds, made up 16.9% and the remaining 1.8% were orphan reads (these could be either due to breaks in the assembly or indicators of possible mis-assembly). A base level comparison of the BAC ends sequenced on the Sanger’s platform with the genome assembly was performed. Out of the 7,783,146 bp in the 11,159 BAC end sequences that aligned to the genome assembly, a total of 7,738,189 bp (99.4%) were found to have exact identity with the genome.

The average GC content of the Asian seabass genome was found to be 41% (S5 Fig). This was compared with a few teleost species and with a representative from each class within the vertebrate subphylum. For both datasets, the 41% value of seabass was found to be intermediate (S5 Fig). Earlier, an inverse relationship between fish genome size and GC content has been observed [20]. Our data are in agreement with those observations, as smaller sized fish genomes (342–463 Mb) showed a higher GC content (44–45%), whereas those with a bigger size (1,010–680 Mb) exhibited lower (37–41%) values. Of the teleost species tested, the zebrafish (*Danio rerio*) had the highest genome size (1.4 Gb) and lowest GC content (36%; S5 Fig).

### Chromosome-level assembly: The first among fish genomes

**Fig 1:**
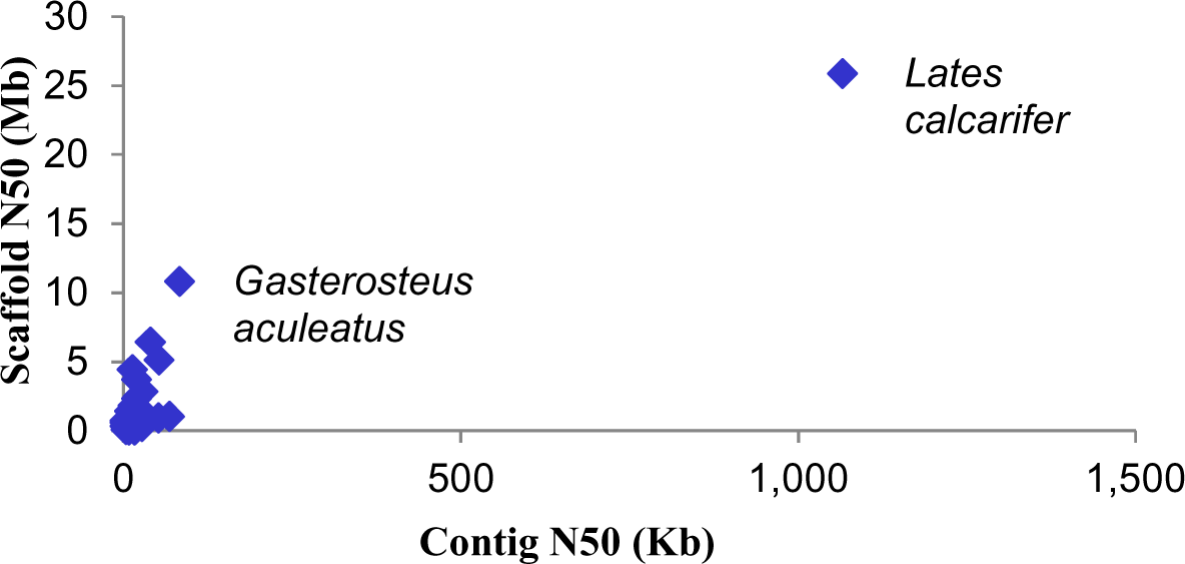
*Lates calcarifer* has the best metrics from among the assembled fish genomes till date. The *L*. *calcarifer* genome contig N50 and scaffold N50 values were compared to the following fish genomes: *Anguilla japonica*, *Astatotilapia burtoni*, *Astyanax mexicanus*, *Boleophthalmus pectinirostris*, *Ctenopharyngodon idellus*, *Cynoglossus semilaevis*, *Cyprinus carpio*, *Danio rerio*, *Dicentrarchus labrax*, *Electrophorus electricus*, *Esox lucius*, *Gadus morhua*, *Gasterosteus aculeatus*, *Larimichthys crocea*, *Latimeria chalumnae*, *Metriaclima zebra*, *Neolamprologus brichardi*, *Notothenia coriiceps*, *Oncorhynchus mykiss*, *Oreochromis niloticus*, *Oryzias latipes*, *Pundamilia nyererei*, *Periophthalmodon schlosseri*, *Periophthalmus magnuspinnatus*, *Salmo salar*, *Scartelaos histophorus*, *Takifugu flavidus*, *Takifugu rubripes*, *Tetraodon nigroviridis*, *Thunnus orientalis*, and *Xiphophorus maculatus* (see S1 Table and S2 Fig for more details).

**Fig 2:**
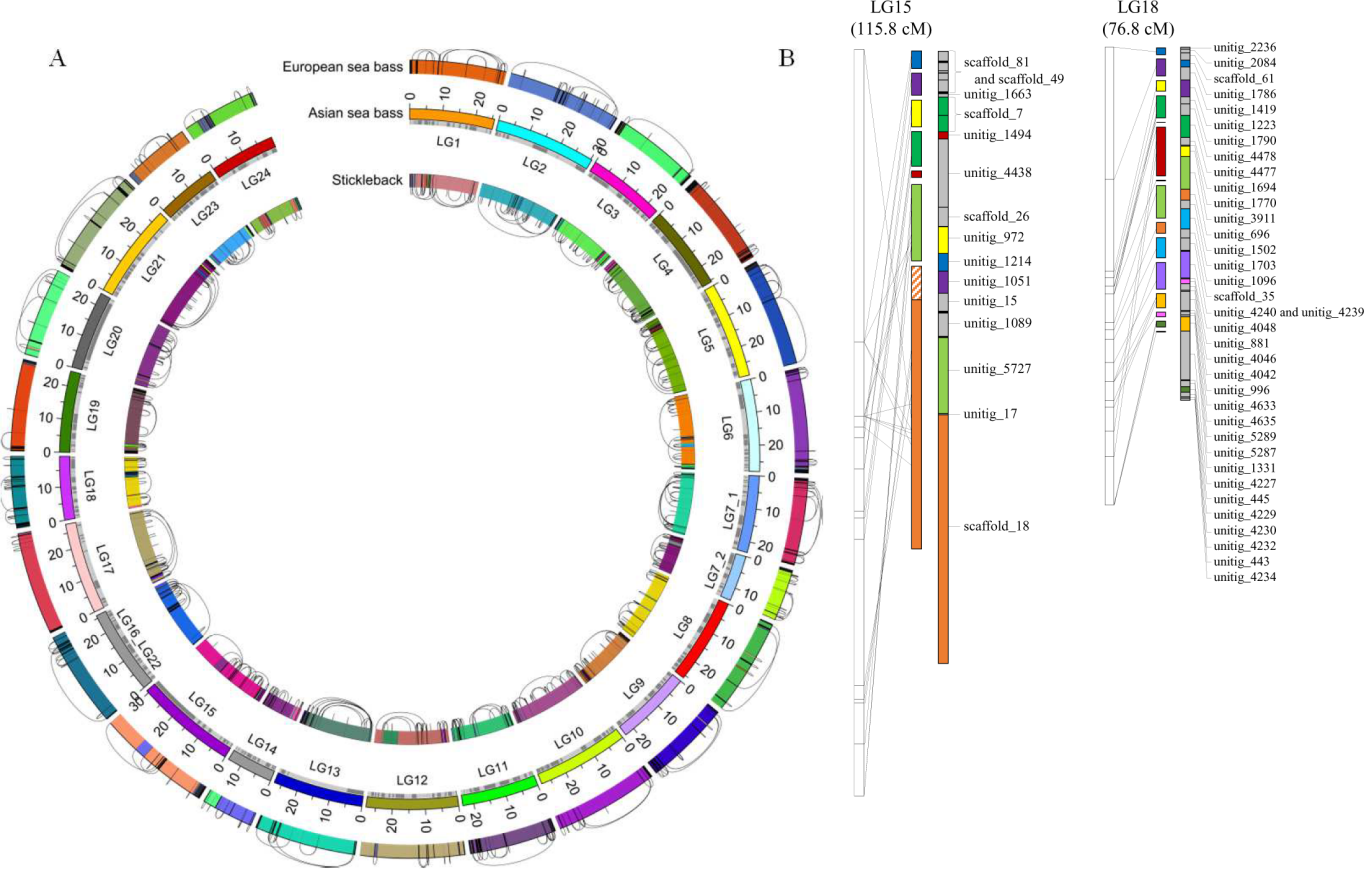
Scaffolding using optical map, genetic map and synteny with closely related fish genomes produced chromosome-level assembly of the Asian seabass genome. (A) Comparison of *L*. *calcarifer* to two closely related fish species (*G*. *aculeatus*, and *D*. *labrax*) at the genome-wide level. Colours used for depicting assembled chromosomes are random for each of the three genomes. Different colours in a single *L*. *calcarifer* linkage group are used to represent the inter-chromosomal rearrangements. Black arcs show collinear blocks that are intra-chromosomally rearranged between the species. (B) Genome assembly (middle panel) shown anchored to two (LG15 and LG18) of the twenty four *L*. *calcarifer* linkage groups while the right panel represents the scaffolded assembly (regions in grey depict the additional contigs brought together by scaffolding).

Nearly 90% of the 772 unique markers described in the Asian seabass genetic map [21] could be anchored to 62% of the assembled genome represented by 24 linkage groups (S11 Fig). Optical mapping was used to scaffold the genome resulting in placement of 73% of the assembled sequences. To further aid scaffolding, the syntenic relationships were compared between *L*. *calcarifer*, *D*. *labrax* [22] and *G*. *aculeatus* [23]. This comparison placed a significant number of smaller contigs into scaffolds that were below the resolution of the optical map. By this approach, the N50 scaffold length could be increased >20 times over the N50 contig length of the primary assembly. Thus, chromosome-level genome assembly (v3; Fig 1) results were obtained upon applying the three methods in the following order: shared synteny; optical mapping and linkage mapping. This order reflects the optimal resolution range (contig sizes that can be placed reliably) of each method [shared synteny (10-100kb), optical mapping (50-200kb) and linkage mapping with several hundred markers (0.5-1Mb)]. Our final assembly involved manual curation, iterative splitting and joining of scaffolds resulting in the construction of chromosomal sequences (see S16 Table for details). By combining all approaches, we were able to place 87% of the assembled contigs into 24 chromosomal scaffolds, having an N50 length of 25.85 Mb and a total length of 587 Mb (Fig 2; S16 Table).

Furthermore, we identified 247 overlaps between ends of neighbouring contigs on the new scaffolds and they allowed us to close 26% of the gaps in the chromosome-level assembly, thereby improving N50 contig size from 1.29 Mb to 1.72 Mb (S17 Table). The resulting genome assembly showed some discrepancies with the lower resolution *L*. *calcarifer* linkage map [21]. To resolve these differences, we split linkage group 7 into two linkage groups (now called LG7_1 and LG7_2), while we combined linkage groups 16 and 22 (now called LG16_LG22). In addition, we revisited the 942 BES pairs which aligned on different scaffolds of the genome by aligning them to the chromosome-level assembly. Of the 942 BES pairs, 566 were found to align to the same chromosome in the correct orientation and size range (50–250 kb).

### Genome-level comparison between three fish species revealed high level of syntenies

We aligned the assembled genomes of *L*. *calcarifer*, *G*. *aculeatus*, and *D*. *labrax*. After filtering for orthologous matches, ~25% of the *L*. *calcarifer* sequence aligned with *G*. *aculeatus* and almost 50% aligned with *D*. *labrax*. We assigned the alignments into syntenic blocks based on shared sequence order and orientation between the query and reference genomes. Syntenic blocks with *D*. *labrax* covered 91.1% of our assembly, with a large N50 collinear block length of about 4.9 Mb. For *G*. *aculeatus*, they covered a similar fraction (90.7%), but the N50 of the collinear block length dropped to 1.8 Mb due to a significantly higher number (367) of intrachromosomal rearrangements spanning >200 kb in the *G*. *aculeatus* genome when compared to *D*. *labrax* (Fig 2). These results show that, similar to other vertebrates, chromosomal synteny in teleost species is well conserved even after >100 million years of evolutionary divergence, despite being considerably more divergent at the nucleotide sequence level (S18–S20 Tables).

### Characterization of repetitive regions and B chromosomes in the Asian seabass genome

The chromosome-level genome assembly (v3) of the Asian seabass genome contains 18.6% repeat sequences (S3 Table), including DNA transposons (5.4%), LINEs (4.0%), LTR (4.6%), retro-elements (2.0%), SINEs (0.3%) and non-LTRs (0.1%) (S7 Table).

**Fig 3:**
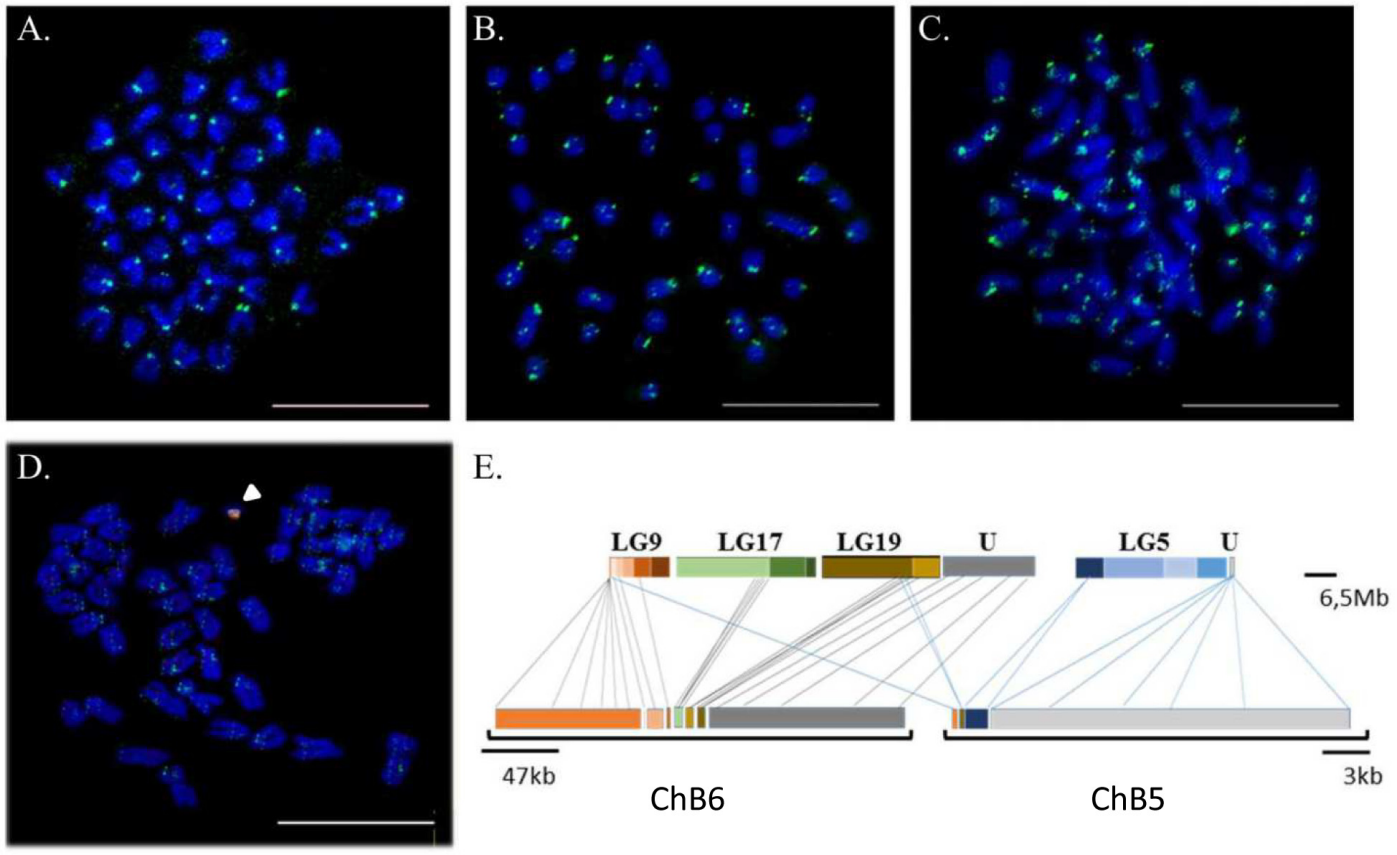
Fluorescence *in situ* hybridization revealed the localization of tandem repeats in centromeric/pericentromeric regions of the Asian seabass genome and characterization of B chromosomes. Labeled painting B chromosomes and tandem repeat probes were hybridized to metaphase chromosomes. The chromosomal position of three tandem repeats (green): (A) Sat_LM- centromeres; (B) Lca_217 and Lca_38 (C) pericentromeric region. (D) B chromosome-derived probes, ChB5 (green) and ChB6 (red), reveal the presence of a B chromosome in the *L*. *calcarifer* karyotype, as indicated by arrowhead. Chromosomes were counterstained with DAPI (blue). Bar is 10 μm for all images. (E) Association of B chromosomes with the linkage groups. Each linkage group is represented in coloured blocks, and the shadings delineate the genome superscaffolds (after optical mapping) that were assigned to the given linkage group. Rearrangements of portions from the four linkage groups, namely LG5, LG9, LG17 and LG19, together with regions without linkage group assignment (U) comprised the B chromosome.

The availability of a highly contiguous assembly allowed for the analysis of the repetitive regions in detail. In total, 11 different types of complex tandem repeat sequences were identified, including the telomeric region, representing 2.1% of the genome (S5 Table). These sequences also included the previously identified OnSat SB [12] and a sequence showing alignment to *Lepomis macrochirus* Sat_LM [24]. Tandem organization of these sequences was confirmed by long PacBio reads allowing us to improve the consensus sequences of these repeats (S1 File). For three of these sequences, primers were constructed and their pericentromeric (Lca_217 and Lca_38) and centromeric (Sat_LM) positions on the chromosomes were identified using Fluorescence *In Situ* Hybridization (FISH; Fig 3). In addition, for four of the 24 linkage groups, the pericentromeric/centromeric location (Lca_217/Sat_LM) was determined (S11 Fig).

In addition to the 24 pairs of A chromosomes, the karyotype of *L*. *calcarifer* contains supernumerary or accessory B chromosomes (AT- and GC- rich) [12]. These B chromosomes were found in variable number in different tissues; with a typical primary fibroblast cell carrying 1–2 DAPI stained B chromosome(s), their size being 5–10% of the average autosome (Fig 3). Three B chromosomes (ChB1, ChB5 and ChB6) were microdissected, amplified and the PCR products used as FISH probes for verification. Most of the ChB1 reads mapped to microsatellite regions with multiple hits in the genome, and hence ChB1 was not used for subsequent analyses. The FISH signals for the B chromosome probes were overlapping with each other (Fig 3). For ChB5 and ChB6, reads that were successfully mapped to the Asian seabass genome were further linked together across 10 kb gap lengths to form pseudo-scaffolds with total length 25,688 bp and 360,387 bp, respectively. Comparison to the genome assembly identified large portions of B chromosomes homologous to LG5, LG9, LG17 and LG19 genomic scaffolds, as well as genomic regions that could not be assigned to specific linkage groups (Fig 3).

### Genome annotation

A total of 22,184 protein coding genes (out of which 90% were located on the assembled chromosomes) were predicted from the masked genome, comprising ~39 Mb of the genome with an average 10 exons per gene (S25 Table). The majority (22,147) of these genes showed a match to a minimum of one InterPro entry (IPR) [25]; 16,671 were associated with at least one Gene Ontology (GO) term [26] and 10,362 were mapped to 350 Kyoto Encyclopedia of Genes and Genomes (KEGG) pathways [27] (S6 Fig and S26 Table). The non-coding RNAs were annotated using the Ensembl pipeline [28]. In total, 2,077 tRNA genes, 3,024 microRNAs, 212 snoRNAs and 1,153 snRNAs were identified. In addition, five small RNA libraries were sequenced from the testis and used to identify 318 high confidence miRNAs, 33 low confidence miRNAs and 51 novel miRNAs (Table 2).

**Table 2:** Annotation statistics of the Asian seabass genome.

Annotation	
Protein-coding genes	22,184
Mean transcript length (bp)	13,448
Mean coding DNA sequence length (bp)	1,737
Mean exons/gene	10
Mean exon length (bp)	170
Mean intron length (bp)	11,714
rRNAs	1,828
miRNAs	3,024
tRNAs	2,077
snoRNAs	212
snRNAs	1,153
miscRNAs	209

In order to inspect the contiguity of the genome assembly, we compared the major histocompatibility complex class I (MHC-class I) genes of Asian seabass with those of the stickleback genome, representing the most complete published fish genome assembly available in the public repositories to date. Fourteen MHC-class I genes were identified in the annotated Asian seabass dataset occupying eight contigs, four of which were >1 Mb in length. By contrast, the MHC-class I genes from stickleback, were located on almost double the number of contigs (14), of which all except one were ≤ 113 kb in length (S14 Fig).

### Phylogenetic analysis and detection of gene duplication events

**Fig 4:**
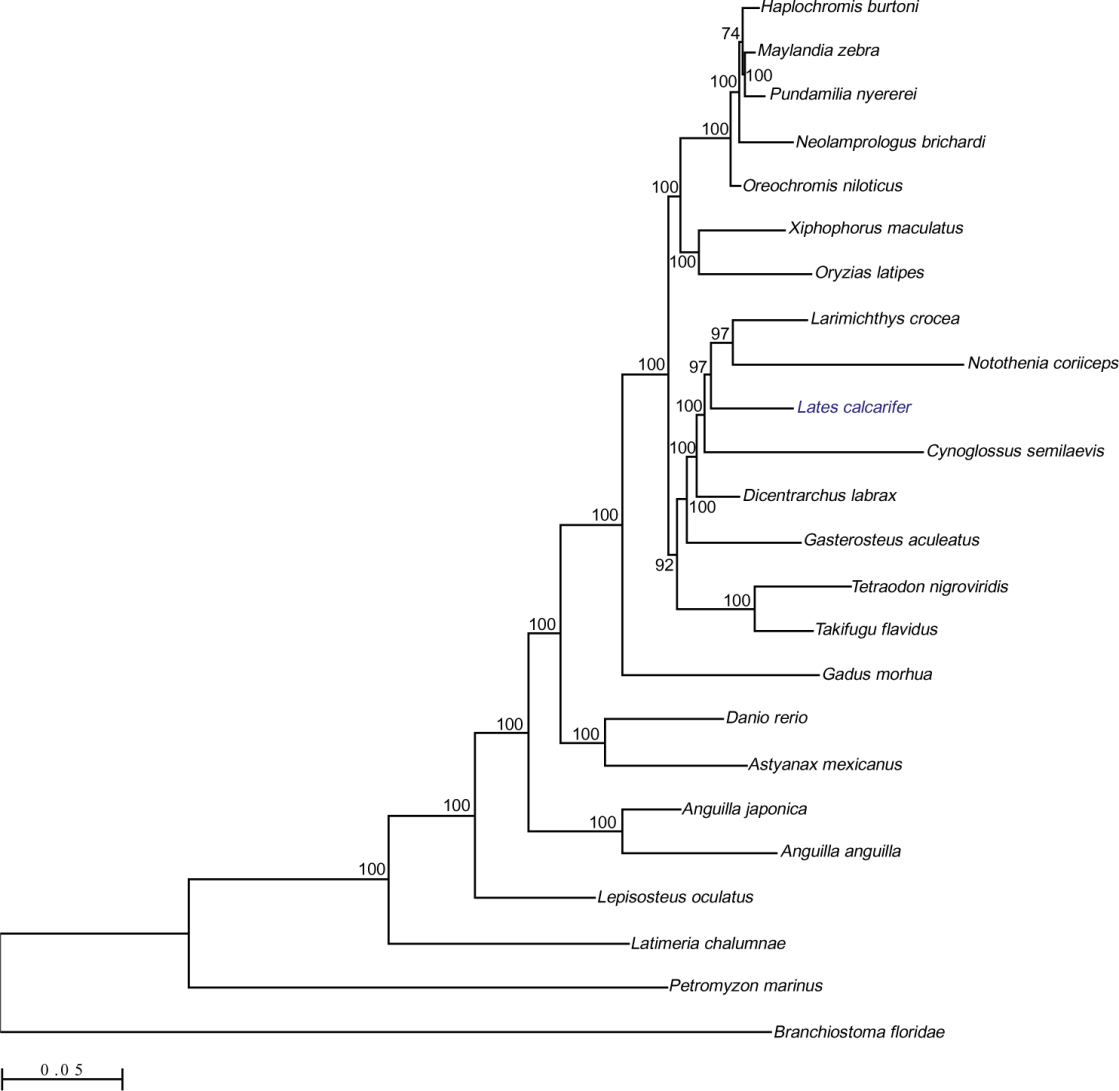
Phylogenetic analyses from 24 species (including 21 ray-finned fishes) depicting the relationship of Asian seab*ass* with the other percomorphs. Maximum Likelihood (ML) tree based on a genome-wide set of 313 strict one-to-one orthologs from 24 species. The concatenated and trimmed alignment spans 127,424 amino acid positions. The scale bar represents 0.05 substitutions per site.

Phylogenetic analyses based on 313 strict 1:1 orthologs from 24 species grouped the Asian seabass with the rest of the percomorph fishes in a well-supported clade (BS 100%, Fig 4). Within this group, our species appeared as a sister group to a clade comprising the yellow croaker (*Larimichthys crocea*) and the cod icefish (*Notothenia coriiceps*; BS 97%). Interestingly, *Cynoglossus semilaevis*, a flatfish, appeared as a sister to the clade comprising *L*. *calcarifer*, *Larimichthys crocea* and *Notothenia coriiceps*.

Teleost-specific genome duplication (TSGD) has created a set of additional gene paralogs in fish genomes and such TGSD-derived gene duplicates have been linked to the evolution of developmental functions in various teleost lineages [29–36]. Using our annotated seabass genome, we set out to identify the potential role of recently duplicated genes towards functional diversification in Asian seabass. A total of 548 duplicate gene pairs were identified following a rigorous process of multiple sequence alignment and phylogenetic tree reconstruction using 20 fish species. These Asian seabas*s*-specific duplicates were enriched for functions critical in immune-modulation, gonad differentiation and glucose transport. Specifically, gene ontology (GO) terms enriched in these duplicated genes (p<0.05) included lipid metabolic processes (GO:0044255; GO:00006629; GO:0046488; GO:0006644; GO:0006650; GO:0009186), threonine-type endopeptidase activity (GO:0004298; GO:0070003), proteosome core complex (GO:0005839), negative regulation of canonical Wnt signaling pathway (GO:0090090), cytokine receptor activity (GO:0004896), interleukin-1 receptor activity (GO:0004908), septin complex (GO:32156; GO:0031105 and Rho GTPase binding (GO:0017048; S30 Table). Metal ion binding functions were not retained in recently duplicated genes of the Asian seabass genome (S31 Table).

### Genomic diversity assessment among *L*. *calcarifer* populations across the native range

**Fig 5:**
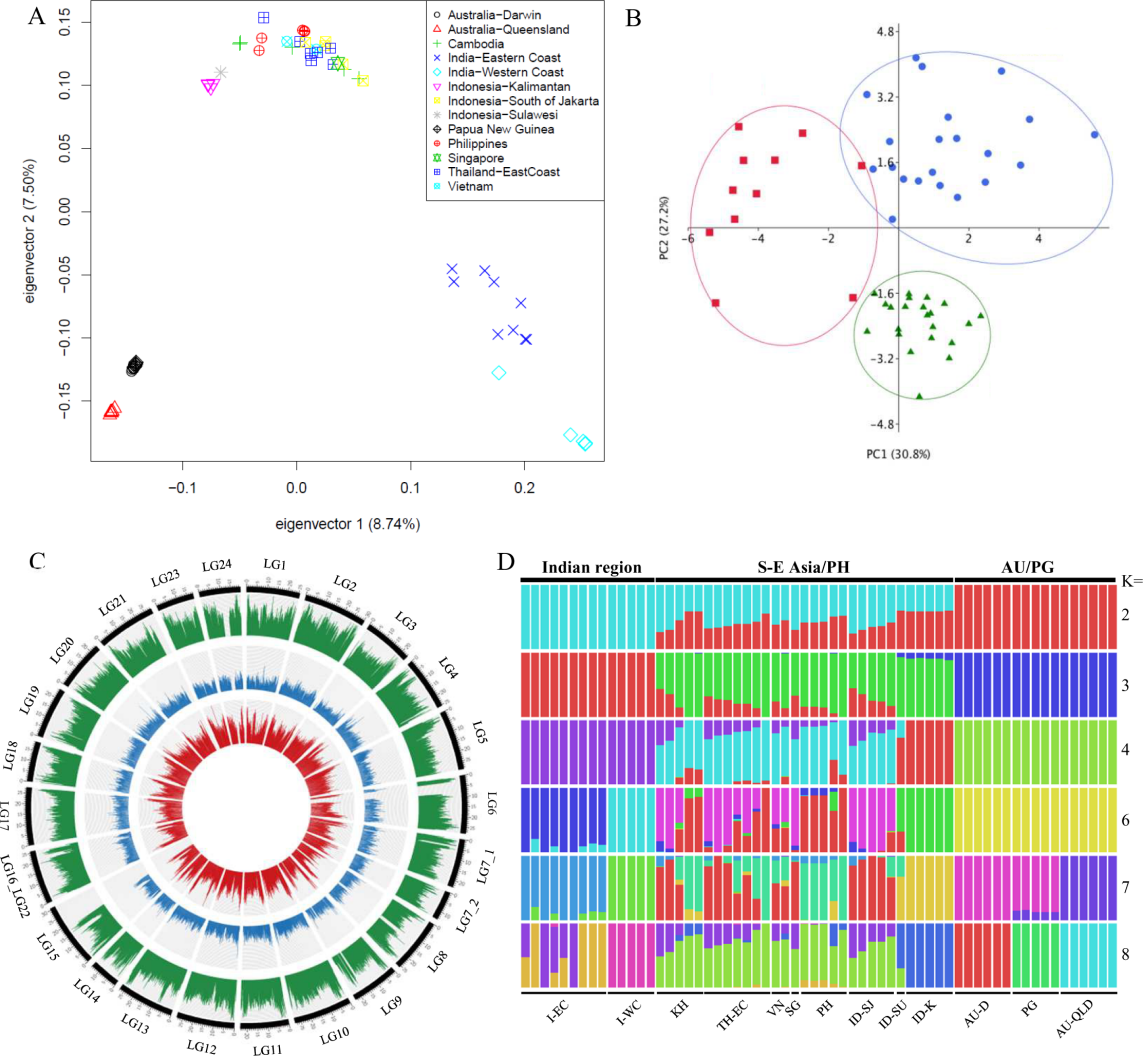
Analysis of re-sequenced genomes supported the existence of *L*. *calcarifer* species complex and its separation into two species (and a third variety). (A) PCA analysis of *L*. *calcarifer* populations using SNPs, (B) PCA analyses using truss morphometric data for representative fishes from the Indian region (red), SE Asia (green) and Australia (blue), (C) Genome-wide nucleotide diversity (Pi) plot representing the three representative species/sub-species of *L*. *calcarifer*. Circos was used to plot nucleotide diversity representing *L*. *calcarifer* from the Indian region (red), Australia/Papua New Guinea (blue), and SE Asia/Philippines (green) based on SNPs in 50 kb non-overlapping windows. The outer scale is 1 Mb. LG refers to the 24 linkage groups of *L*. *calcarifer* with the discrepancies identified in the process of genome scaffolding reflected in the altered IDs for certain linkage groups (LG7 split into two—LG7_1 and LG7_2; LG16 and LG 22 combined- LG16_LG22) and (D) Admixture analyses showing iterations from K = 2,3,4,6,7,8. Each individual is represented by a vertical bar. Abbreviations: I-EC (India-Eastern coast), I-WC (India-Western coast), KH (Cambodia), TH-EC (Thailand-Eastern Coast), VN (Vietnam), SG (Singapore), PH (Philippines), ID-SJ (Indonesia-South Jakarta), ID-SW (Indonesia-Sulawesi), ID-K (Indonesia-Kalimantan), AU-D (Australia-Darwin), PG (Papua New Guinea), AU-QLD (Australia-Queensland).

Low coverage genome re-sequencing was performed on 61 individuals from 12 diverse locations on the Illumina platform (6.7X average sequencing depth; S13 Fig and S22 Table). This, together with the genome sequence information of the individual used to produce the reference genome, was used to assess the genetic diversity within the *L*. *calcarifer* species complex and to facilitate the identification of polymorphisms associated with useful traits such as growth and disease resistance (Fig 5A–5D). The sampling represents the native range of the species, extending from North-Western India, through SE Asia to North-Eastern Australia (S21 Table). With the exception of Philippines, Vietnam and Singapore, all individuals from the remaining regions were wild-caught. In total, 5,642,327 SNPs with Phred quality >30 were identified. Three groups (Indian region, SE Asia/Philippines, and Australia/Papua New Guinea) bearing clear allopatric signatures of separation could be observed through Principal Component Analysis (PCA) based on SNPs. Although samples from the Indian region and Australia/Papua New Guinea represented distinct clades, the individuals from SE Asia/Philippines showed signs of admixture with fishes from Kalimantan and Sulawesi being more divergent compared to the remaining fishes from SE Asia and Philippines (Fig 5D). Similar results were obtained through phylogenetic analyses (S10 Fig). The genome-wide nucleotide diversity (Pi) plot (Fig 5C) for the three identified groups of *L*. *calcarifer* similarly demonstrated high level of nucleotide diversity in the SE Asian/Philippines group whereas individuals from Australia had the lowest level of diversity and those from the Indian region showed moderate genome-wide polymorphism. Admixture analyses further revealed that the majority of individuals grouped together on a micro-geographic scale within regions, suggesting a degree of evolutionary philopatry within the species (Fig 5D**).**

In addition, morphometric analysis was performed to establish whether there were discernible phenotypic differences between the fishes representing the three regions (Indian, SE Asian and Australian). PCA based on truss measurements (normalised by maximum length of fish) also supported the existence of three distinct populations of *L*. *calcarifer* (Figs 5B, S12 and S13; S24 Table). Further, of the 18 truss measurements studied, the use of V3 (point on dorsal surface of the fish that is exactly perpendicular to the base of pectoral fin to the anterior base of the dorsal fin) and V18 (dorsal base to ventral base of caudal fin) [8] could confidently classify 73.8% of the fishes to their respective groups of origin. The classification accuracies were high for individuals from Australia and low for those from the Indian region with fish having higher V18 value most likely to be classified as fish from Australia.

## Discussion

Due to TSGD, fish genomes tend to contain more gene paralogs than those of other vertebrates [37–39]. This results in the unmatched diversity seen in fishes, the most diverse group of vertebrates, represented by more than 34,000 species [40].

Of the fish genomes published till date (S23 Table) [41], many have been driven by enquiry into the evolution of fish genomes, chiefly stemming from the TSGD event and the resulting additional repertoire of genes, as in the case of cichlid genomes [42], three-spined stickleback [23], Japanese medaka (*Oryzias latipes*) [43], and green spotted pufferfish (*Tetraodon nigroviridis*) [44]. Fish genomes have also been sequenced for their value as a research model for vertebrate/human disease, e.g. zebrafish [20] and platyfish (*Xiphophorus maculatus*) [45]. Our motivation to sequence the Asian seabass genome stemmed from the fact that the species is rapidly becoming important from an aquaculture perspective. An improved understanding of the genome will help in the implementation of molecular information into breeding programs, similar to the Atlantic cod (*Gadus morhua*) [46], European sea bass (*Dicentrarchus labrax*) [22], salmonids (rainbow trout (*Oncorhynchus mykiss*) [47], Atlantic salmon (*Salmo salar*) [48]) and tongue sole (*Cynoglossus semilaevis*) [49].

The majority of the eukaryotic genomes published to date have been assembled using short read sequencing technologies. Our approach represents a change in this trend wherein the assembly is based solely on long reads obtained on the PacBio’s SMRT technology [13,14]. This strategy seems ideal for assembling mid-to-large eukaryotic genomes since it ensures contiguity, less ambiguity and assembly metrics surpassing all of the fish genomes sequenced thus far. With advances in technology development, the latest chemistry from PacBio can produce average read lengths of 10–15 kb, implying that eukaryotic genomes surpassing the metrics reported in this work can be expected in the near future. Although the genome information was obtained from a heterozygous individual, a diploid unaware assembler (Celera used as part of HGAP [15]) was used for assembling the genome, therefore, it was not possible to phase the variation between the maternal and paternal chromosomes.

Although the initial *de novo* assembly had outstanding metrics, we integrated optical mapping to improve the assembly further. The optical map and the primary genome assembly (v1) were in excellent agreement with only 55 discordances identified (S15 Table). Optical mapping thus served as an independent validation for the assembly and additionally yielded information to extend and obtain a chromosomal level assembly (scaffold N50 >25 Mb) of the *L*. *calcarifer* together with integrating genetic linkage map data and evolutionary evidence. This chromosome-level genome assembly will accelerate the development of genomic platforms to improve the aquaculture of the species and it will also allow better understanding of fish genome structure and evolution.

**Fig 6:**
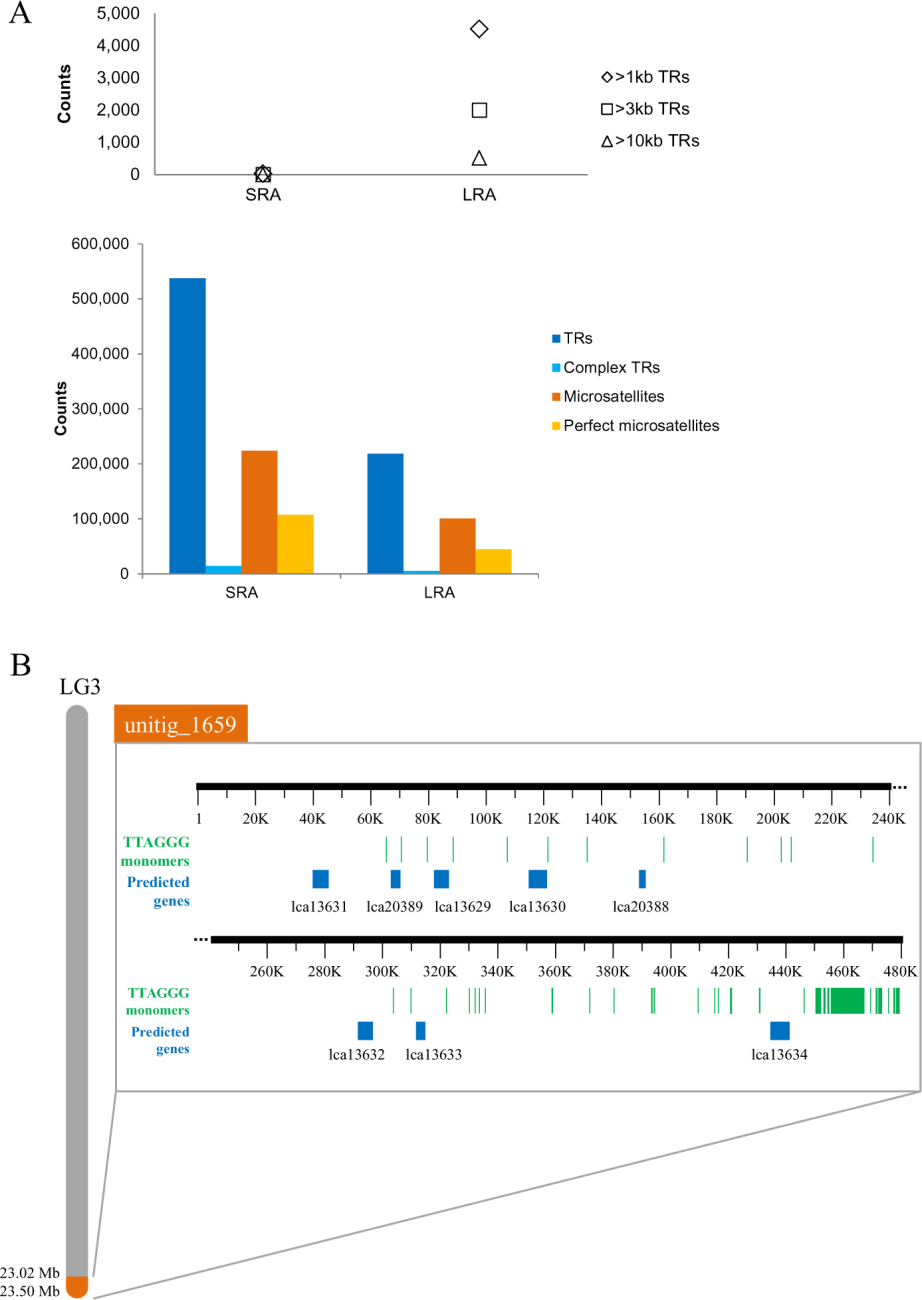
Survey of the *L*. *calcarifer* genome assembly identified long stretches of TRs lacking in the short read-based assembly and a continuous assembled telomeric region identified at the end of LG3. (A) Stretches of TRs were virtually missing from the *L*. *calcarifer* short read assembly (SRA) generated using 80X Illumina reads scaffolded with ~11,000 BAC ends (S1 Table) whereas the long read assembly (LRA) had a good representation of TRs (upper panel) and the different repeats were more fragmented in the SRA *vis-à-vis* the LRA (lower panel). (B) Arrangement of telomere monomer sequence (TTAGGG) on a single assembled contig, (unitig_1659; ~0.5 Mb) placed at the terminal end of LG3 (region indicated in orange). Every occurrence of the monomer is indicated by green bars. A highly dense region of (TTAGGG)n was observed between 455.5–466.9 kb, containing the monomer repeated in tandem 1,655 times. The region upstream to this dense region had short dispersed stretches of (TTAGGG)n and contained eight predicted genes (indicated by blue boxes).

Mobile elements are considered the primary drivers of genome expansion [50]. Several conserved fish retrotransposons, such as MAUI [51], Gypsy [52], Rex [53], Bell and TART [54] could be identified in the assembled seabass genome (S6 Table). All of these elements were represented in the transcriptome [12], and were also found in the predicted gene set indicating the likelihood for them to be active in the genome. Tandem repetitive sequences form the core component of centromeres and telomeres and thus represent the most complex part of eukaryotic genomes. However, they are difficult to capture and assemble due to forming continuous arrays of tandemly repeated monomers, and are therefore, rarely contained in most genome assemblies. The long sequencing reads spanned repeat-rich regions, providing an opportunity to resolve and characterize many tandem repeat regions in the Asian seabass genome [12]. Also a number of different classes of tandem repeats were used to estimate assembly quality (Fig 6A). The primary assembly (v1) based on 90X PacBio coverage contained the largest fraction of tandem repeats (10.54%) including 516 arrays greater than 10 kb that makes it comparable to the human genome assembly which contains 503 arrays of tandem repeats >10 kb [55]. On the other hand, the assembly based on short Illumina paired-end reads (Fig 6A) had only two assembled arrays more than 3kb.

We identified the standard telomeric repeat (TTAGGG)n [56] in a ~478 kb contig at the terminal end of LG3 that contains a dense region of repeat units in tandem. Upstream to this dense region, the telomeric repeats were dispersed and intersected with eight predicted genes (Fig 6B). Such kind of structures are similar to the varied telomere array organization observed in chicken [57], *Chironomus pallividittatus* [58], fruit fly [59], and human [60] genomes. Conversely, in the case of Illumina assembly stretches of telomeric monomers repeated for >100 bp could not be identified. In addition, monomers of centromeric sequences (Sat_LM) identified in our sequenced dataset were organized similar to the telomeric sequences. They were found as a ‘train of’ 2–6 monomers with head-to-tail organization and interrupted by various short fragments of transposon elements with the exception of Lca_217 (a peri-centromeric repeat element) that demonstrated a higher-order organization that is usually observed for pericentromeric regions.

B chromosomes or accessory chromosomes occur in approximately 15% of eukaryote species and typically represent almost 5% of the genome [61]. The Asian seabass B chromosomes were found to be mosaics of different autosomal chromosomes; similar to what has been reported for other fish B chromosomes [61,62]. In addition, the centromeric and pericentromeric regions typically associated with autosomal chromosomes were lacking from the heterochromatin of the B chromosome analyzed.

One of the most interesting aspects of the biology of *L*. *calcarifer* is the ability to change its sex from male to female following maturation [3–5]. We have been studying the process of sex change using zebrafish–a species, where males undergo a female-to-male gonadal transformation during their development–as a model [63–68]. Recent data about the pathways regulating the natural sex reversal appear to indicate conservation among sex changers independently from the direction of the change [69]. The reference genome and transcriptomic data (both coding and non-coding) produced from gonads at different stages of their transformation are expected to be useful for ongoing research on the sex reversal process. Interestingly, Asian seabass-specific gene duplicates were enriched for functions related to gonad development. This included three genes with increased number in the seabass compared to other teleosts: *anti-mullerian hormone (amh)*, *nuclear factor κβ 2* (*nfkb2*), and *septin 7 (sept7)*. Of these, the first two have been shown to play a role in the gonadal transformation of zebrafish [64,68,70] and exhibited differential expression between the male and female gonads of the seabass [69]. The potential role, if any, of the duplicated genes in the sex reversal process will have to be further investigated. As sequential hermaphrodites need to retain the ability to change their sex after maturity, they do not have a classical, sex chromosome-based sex determination system. Those genes whose product pushes the system towards either of the two sexes are scattered throughout the genome and therefore such species could become excellent models for polygenic sex determination [63].

*L*. *calcarifer* occupies a broad geographic native range extending from the Western coast of India to Northern Australia. There is considerable genetic diversity in the population, mostly attributed to the apparent geographic barriers to reproduction [8]. In fact, even within Australia, three major populations are recognized that include those from Western Australia, Eastern Australia, and a central admixed population [71]. Analyses of the genetic diversity through the natural range of *L*. *calcarifer* using low-coverage whole genome re-sequencing confirmed earlier indications (based on mitochondrial markers) [8,72] for three separate cohorts and revealed a clear allopatric demarcation of individuals representing the three regions comprising: India; Australia/Papua New Guinea; and SE Asia/Philippines. Of these, only the first two groups displayed distinct micro-geographic separation amongst the regions representing the whole group reflecting limited mixing between the Indian and Australian populations. The low level of genetic diversity observed in the Australian populations is possibly due to a founder effect. This is in agreement with our previous observation of signs of hybridization (with the Indian region fishes) being present within the wild type population from Malaysia, Thailand and Indonesia whereas, wild SE Asian fishes could not be identified in the Indian population [8]. Thus, the clear signs of admixture and high level of genomic diversity seen in the SE Asian population are due to secondary contacts, translocation and hybridisation with the Indian population. This pattern can possibly be attributed to unrecorded translocation of fishes from the Bay of Bengal/Andaman Sea to the South China Sea or due to migration, though its breadth needs to be established [8,73]. Thus, both nuclear and mitochondrial genome sequences point to *L*. *calcarifer* existing as a species complex attributed to an allopatric *species-split*, with *L*. *calcarifer* from the Indian region representing a species distinct from the fishes from SE Asia, with fishes from Australia and Papua New Guinea forming a sub-group within the latter. The whole genome sequencing and re-sequencing help to resolve the population structure of the species and facilitate the analysis of genetic diversity. The data will be useful for developing genomics-based assays such as allele mining, genomic selection, genotyping by sequencing and genome-wide association studies (GWAS), providing the impetus for the aquaculture production of the species, currently prevalent in the Indo-West Pacific and increasingly being introduced to several other countries such as Saudi Arabia, Iran, Guam, French Polynesia, USA and Israel [1].

## Methods

### Ethics statement

Farmed Asian seabass (*Lates calcarifer*) were obtained from the Marine Aquaculture Centre (Singapore). All experiments were approved by Agri-food and Veterinary Authority (AVA) Institutional Animal Care and Use Committee (IACUC) (approval ID: AVA-MAC-2012-02) and performed according to guidelines set by the National Advisory Committee on Laboratory Animal Research (NACLAR) for the care and use of animals for scientific research in Singapore.

### Choosing the individual for the Asian Seabass Genome Project

Backcrossed and inbred fish were genotyped to identify the fish which exhibited the least heterozygosity. Individuals displaying a range of 5–50% homozygosity could be identified based on the markers used for genotyping [10]. A backcrossed individual (BC-8) with decreased genomic polymorphism (DGP; ~40% based on the markers analyzed) was chosen and sacrificed to collect blood, as well as the majority of organs to serve as a resource for the Asian Seabass Genome Project.

### Genome size and heterozygosity analysis

We estimated the genome characteristics using a k-mer analysis of the raw Illumina sequencing data and estimated the genome wide rate of heterozygosity by evaluating the shape of the k-mer frequency distribution.

We counted the frequency of all k-mers (k = 21) in the data using Jellyfish [74]. The observed k-mer distribution was bimodal with distinct peaks at ~36x and twice this amount at ~72x coverage (S1 Fig), which is characteristic for a heterozygous genome [75]. We analyzed the distribution by fitting a mixture model of two negative binomial distributions centered at *mu* and mu*2, representing the heterozygous and homozygous k-mers, respectively (Eq 1). The negative binomial components generalize the Poisson distribution with an additional *size* parameter for the variance. The k-mer model also includes two scaling parameters *s*_*1*_ and *s*_*2*_ that depend on the genome size and the overall rate of heterozygosity.

$$K\;\left(x\right)=s1*dnbinom\;\left(x,mu*size_{1}\right)+s2*dnbinom\;\left(x,mu*size_{2}\right)$$ (1)

**Eq 1.** k-mer coverage model for heterozygous diploid genomes.

We determined the parameters of the model using a nonlinear least-squares estimate with the *nls* function in R. The resulting values fit the data well, with the two peaks centered at 36.6x and 73.2x coverage and small residual error (S1 Fig). This also naturally excludes the low coverage k-mers caused from sequencing error that should not be included in the genome size or heterozygosity estimates. The modeling allows us to estimate the number of non-repetitive heterozygous and homozygous k-mers as 96,126,339 and 491,559,122, respectively, by summing the densities for the two components of the model separately. Using these values, we further estimate the overall rate of heterozygosity to be 0.4%-0.5% (Eq 2).

$$Rate\;of\;Heterozygosity=\frac{\left(\#\;het.\;kmers\right)/\left(2*k\right)}{\left(\#het.\;kmers\right)/2+\;\#homo.kmers}$$ (2)

**Eq 2.** Formula to determine rate of heterozygosity.

The number of heterozygous k-mers in half for both the numerator and denominator is divided in half to evaluate the haploid content. The number of heterozygous k-mers is divided by k to account for that heterozygous base will contribute k heterozygous k-mers (k = 21). This will slightly undercount heterozygous bases that are within K bases of each other, allowing for a range in the overall heterozygosity rate.

### Extraction of genomic DNA and generation of BAC libraries

For generating genome sequence data, genomic DNA was isolated from the chosen individual using Qiagen GenomicTip100 (Qiagen, Hilden, DE) as per standard protocol. Genomic DNA from the blood was used for the construction of two separate BAC libraries in pCC1BAC vector (Amplicon Express, Pullman, WA, USA) altogether representing 12X genome coverage. Libraries comprised an *Eco* RI (36,864 clones) library with an average insert size of ~120 Kb and a *Bam* HI BAC library (36,864 clones) with an average insert size of ~115 Kb.

### Genome sequence data

Sequence data for the genome project was obtained from multiple platforms which are summarized below:

#### Single-molecule, real-time sequencing system (SMRT) data

Sequence data from the PacBio platform was generated in two phases:

- Asian seabass brain genomic DNA (10 μg) was used for generating a single ~10 kb insert size library which was sequenced using 77 SMRT cells (1X120 minutes movie) on PacBio RSII using C2-XL chemistry (DNA Link, Korea). The average data per SMRT cell was 264 Mb (30X coverage). An average read length of 4,498 bp and average base quality score of 0.83 was obtained for the sequence data. A total of 20.3 Gb of data representing ~30X coverage of the Asian seabass genome was generated.
- Asian seabass kidney genomic DNA was used for generating a ~20 kb insert size library which was sequenced using 105 SMRT cells (1x180 minutes movie) on PacBio RSII using P5-C3 chemistry (DNALink, Seoul, Korea). All DNA was size selected (>7 kb) using the Blue Pippin system (Sage Sciences, Beverly, MA, USA) and samples were sheared with g-TUBEs (Covaris, Woburn, MA, USA)). The average data per SMRT cell was 421 Mb (60X coverage). An average read length of 8.09 kb and average base quality score of 0.83 was obtained for the sequence data. A total of 42.14 Gb of data or a ~60X coverage of the Asian seabass genome was generated.

#### Whole genome shotgun sequencing using Illumina HiSeq 1500 Platform

Asian seabass liver genomic DNA from the chosen individual was used for the construction of two (500 bp and ~750 bp) linear insert TruSeq (Illumina) libraries (as per standard protocol) and sequenced using HiSeq 1500, with one lane for each insert sized library (2X100). A total of 280 million pairs of reads were generated (~56 Gb data), representing 80X coverage of the Asian seabass genome.

#### BAC library end sequencing details

The first 7.5 plates (each containing 384-wells) from each library (*Bam* H1 and *Eco* R1) were sequenced from both ends using M13 forward and reverse primers. The overall data quality of sequences was good, with a pass rate of ~95% and an average of 769 high quality (Phred20) bases per read. The total number of trimmed sequences from *Eco* R1 and *Bam* H1 library (Forward + Reverse) was 5,703 and 5,646, respectively, making a total of 11,349 sequences.

### Primary de novo genome assembly (v1; 90x PacBio data)

The primary genome assembly with 90x PacBio data was performed using HGAP algorithm followed by polishing using Quiver [15]; as part of the Pacific Biosciences SMRTAnalysis pipeline. To facilitate genome assembly, a *make* script available at https://github.com/pbjd/smrtmake was used. The statistics of the 90X PacBio assembly are detailed in S1 Table.

### Additional genome assemblies

#### De novo genome assembly - 80X Illumina data and BAC end sequences

QUAKE [76] and decontamination was performed on the Illumina Hiseq reads and assembled along with BAC end sequences using SOAPDenovo127mer [77] (k-mer was set from 21–81). The statistics are summarized in S1 Table.

#### Alternative PacBio genome assembly

For the assembly done at CSHL, instead of using the full Celera assembly pipeline [78], the algorithms were used separately. BOGART [79] was used for unitigging (to help resolve some of the repeat issues). PacBio’s HBAR-DTK [15] was used to finalize the assembly. The HBAR-DTK software retained the singletons (unlike the Celera assembler [78] which discards singletons and only keeps consensus contigs). There were totally 3,604 singletons in this assembly and after their removal, the number of contigs reduced to 4,223, the assembly statistics are summarized in S1 Table.

### Use of transcriptome data for genome scaffolding

The assembled seabass transcriptome [16] was used for scaffolding the 90X PacBio genome assembly using the L_RNA_scaffolder tool [17] (S1 Table) resulting in the scaffolded genome assembly (v2).

### Assessment of genome assembly quality

The assembled genome was evaluated using different metrics described below:

#### CEGMA- and QUAST-based evaluation

The assembled genome was evaluated for completeness using the 248 core eukaryotic genes dataset (CEGs)[18,19] and for quality using QUAST [80].

#### Validation of the genome assembly using BAC end sequences (BES)

The 11,191 BAC End Sequences were used for aligning against the genome assembly (BLASTN [81], minimum alignment length cut-off of 100bp and a threshold e-value of 1e-6). A base level comparison was also done wherein 7,783,146 bp bases representing 11,159 BES were compared (using BLASTN [81]) to the genome assembly.

#### Validation of the genome by mapping Illumina PE genome reads to assembly

For both the 500 and 750 bp Illumina libraries (totaling to ~80X genome coverage), >99% of the HiSeq paired-end reads mapped correctly to the genome assembly with >97% of the reads in pairs with similarity cut off of 90% and aligned read length 90% (S3 Fig).

### Anchoring the genome assembly to linkage groups

A total of 772 unique marker sequences from the Asian seabass linkage map [21] were BLAST-searched [81] against our genome scaffold sequences, retaining the top five BLAST hits for further analyses. For each marker on the linkage group, we selected the best alignment based on marker sequence alignment coverage. If a marker sequence had more than one alignment to different regions of the genome, but with the same alignment coverage, both alignments were retained. Further to that, only alignments that had percentage identity ≥90% and marker alignment coverage ≥80% were kept. Finally, if a given genome scaffold had markers from multiple linkage groups aligned to it, a manual inspection was performed to select for the linkage group that was represented predominantly. This analysis resulted in 680 (88%) of the marker sequences having an alignment to the genome scaffolds (as shown in S11 Fig).

### Scaffolding using optical mapping (Opgen)

Optical map data generation and the whole genome *de novo* assembly process have been described in detail earlier [82–84]. Briefly, high molecular weight (HMW) DNA was obtained from frozen blood of the same Asian seabass individual from which genome sequence information was obtained using OpGen’s blood processing protocol (OpGen, Gaithersburg, MD, USA). The resulting DNA was evaluated on OpGen’s Argus Whole Genome Mapping System and quality metrics were reviewed. The average molecule size obtained from this DNA prep was approximately 304 kb. *XbaI* was selected as a suitable enzyme for generation of the optical map data. A total of 11 high-density MapCards were selected from those generated by Argus. On an average, ~68,000 Single Molecular Restriction Maps (SMRM) were marked up on each card. Typically, only molecules longer than 250 kb (total 377,118 SMRMs) were used in the analysis. These SMRMs were assembled into genome-wide Maptigs by using OpGen’s Gentig software [85,86] by aligning SMRMs based on restriction map pattern using a greedy algorithm with limited backtracking for finding an almost optimal scoring set of Maptigs. Gentig also takes into consideration the possible errors of SMRMs such as standard deviation, digestion rate, false cut and missing cut during the assembly process. Totally, 104 Maptigs representing the whole genome of Asian seabass were generated. These assembled Maptigs were then aligned with scaffolds of the assembled genome sequences (≥40 kb) to aid in the orientation and joining of these sequence scaffolds, resulting in the assignment of 577 contig sequences with a combined length of 486.38 Mb (S9–S14 Tables).

The optical map data also identified 55 sequences that potentially contain mis-assemblies, as different parts of these sequences showed alignment onto different Maptigs (S15 Table).

### Chromosome-level assembly of Asian seabass by integrating data from four platforms

We first performed whole genome alignment of the primary Asian seabass genome assembly (HGAP contigs) with the chromosomal scale genome assemblies of European seabass [22] and three-spined stickleback [23] using the LAST alignment tool [87]. The output MAF files were filtered for 1:1 ortholog alignments using single_cov2 [88]. Subsequently, we combined pairwise alignments into multiple alignments using the multiz tool [89]. We then used Ragout [90] to infer the order of Asian sea bass contigs according to colinearity with *G*. *aculeatus* and *D*. *labrax* assuming a closer relationship of *D*. *labrax* and *L*. *calcarifer*. The ordered contigs were written into scaffold sequences and in a second iteration aligned with the *D*. *labrax* genome alone, which enabled us to find further contig links. After each iteration, the resulting order of contigs was manually checked to remove suspicious interchromosomal connections. Subsequently, we compared the results with data from optical mapping and removed contig links that were clearly not in agreement with the contig order from optical mapping. Resulting scaffolds that were supported by shared synteny and optical mapping were relatively large, contained most of the assembled *L*. *calcarifer* sequence and could be ordered into chromosomal sized sequences using information from the *L*. *calcarifer* genetic linkage map [21].

We performed BLASTN [81] alignment (min. alignment identity 95%) between neighbouring contigs (a,b) placed in the assembled chromosomes and found a large number of contig end (a) to contig start (b) overlaps (only +/+ strand overlaps were used). Gaps between overlapping contigs were then closed by trimming the overlap region from the contig (a) and concatenating contig (b) to it. This process was performed by custom scripts written in Linux AWK language (S16 and S17 Tables).

### Chromosome-level assembly comparisons between *L*. *calcarifer*, *D*. *labrax* and *G*. *aculeatus*

As described above, whole genome alignments of the final chromosome scale assembly (v3) of *L*. *calcarifer* with *D*. *labrax* and *G*.*aculeatus* genomes were performed by LAST [87] and filtered by single_cov2. MAF output files were subsequently converted to the Satsuma format by custom scripts (AWK) and processed by the BlockDisplaySatsuma script from the Satsuma v1.17 package [91] to result in coordinates of syntenic blocks between the different genomes. This process was done in two iterations. The removal of spurious very short blocks (<6000 bp) after iteration 1 resulted in larger collinear Blocks after iteration 2. Syntenic blocks along the 24 *L*. *calcarifer* chromosomes were plotted using CIRCOS [92]. Additionally, we plotted links between collinear blocks to underline if they were rearranged in *D*. *labrax* or *G*. *aculeatus*.

### Repeat masking and inventory

The assembled Asian seabass genome was masked based on known repeats using Repeat Masker [93] and the RepBase vertebrate libraries [24] (S2 Table). *De novo* repeat masking was performed on the genome with WindowMasker [94] and RepeatScout [95].

A collection of tandem repeats were obtained using Tandem Repeat Finder (TRF) version 4.07 [96] and post-processed [97]. The parameters used for the TRF search were: maximum mismatch 5; maximum period size 2000, and default values for other parameters (S3 Table).

Miniature inverted-repeat transposable elements, LTR elements and potential transposon ORFs were also determined from the whole genome assembly using MITE Hunter [98], LTR-harvester [99] and TransposonPsi (http://transposonpsi.sourceforge.net/), respectively. The predicted genes from the Asian seabass genome were later searched against transposable elements obtained from the LTRharvest, Mites and TransposonPSI databases (e-value of 1e-06 and 80% percentage alignment length) to remove predicted genes that aligned with transposable elements. Transfer RNAs (tRNAs) were also searched by tRNAscan-SE [100].

### De novo RepeatScout library characterization

The consensus sequences for repetitive families generated by RepeatScout (8,248 repeat families; see S6 Table for details) were classified using TEclass [101] into four categories according to their mechanism of transposition, namely, DNA transposons, LTRs, LINEs and SINEs. Consensus motifs that showed sequence similarity to RefSeq [102] genes were filtered out, as they are likely to belong to a gene family, or be part of a conserved domain. To assign a repeat classification to the consensus motifs of repetitive regions, they were searched against the transposable elements determined from the genome assembly by MITE Hunter, LTR-harvester and TransposonPsi as well as the repeats present in RepBase [24].

### Obtaining tandem repeats from 23-mer HiSeq reads

We used the Jellyfish software [74] for computing 23-mer frequencies and choosing a subset of 23-mers with coverage greater than 1,000. We used the Cookiecutter package [103] for extraction of raw reads containing subset of 23-mers with coverage greater than 1000. The selected reads were used to manually assemble tandem repeat monomer consensus sequences with the help of the targeted *de novo* short-read assembler PRICE [104].

Assembled tandem repeats were compared with known Repbase repeats [24] and all related to transposable elements were excluded. Following which, tandem repeats assembled from raw reads were aligned against the PacBio error corrected reads using BLAST [81] to improve the consensus sequences by including more individual monomers. The consensus sequences were used to find and estimate the repeat copy number and arrangement in the genome assembly. The interruption of tandem repeats monomers arrays in SMRT reads could be verified by HiSeq reads containing transition fragments.

### Generating the protein-coding gene set

#### Masking low complexity regions

The genome was screened using RepeatMasker (version 4.0.5) [93] against the entire vertebrate repeat library and subsequently against the published *L*. *calcarifer* specific repeats [12]. Interspersed repeats were hard-masked with Ns and simple repeats were soft-masked. A final round of masking was performed using DustMasker (RMBlast, version 2.2.28) [105].

#### Protein coding gene predictions

First, putative gene loci were identified. The “other vertebrates” protein collection from Genbank [106] was retrieved and filtered to retain only proteins from ray finned fish species. The resulting 388,340 proteins as well as the 1,184,879 reconstructed transcripts and ESTs were mapped to the genome using a tblastn/blastn run through GenBlastA [107], which provided coordinates for putative gene loci.

Protein/transcript alignments were then refined and Augustus hints were generated. The genomic region of the putative gene loci (+25 kb flanking regions) were excised, and the corresponding protein/transcript sequences, linked to these gene loci by GenBlastA, were aligned using exonerate [108]. Customized scripts were used to convert the exonerate output to GFF3 format with genomic coordinates, and generate an Augustus hints file.

A *L*. *calcarifer*-specific training annotation file was generated through the Augustus training web interface [109] using 22,322 *L*. *calcarifer* ESTs. Augustus was run separately for each genomic contig using default parameters, extrinsic.E.XNT.cfg, the contig-specific hints file and the *L*. *calcarifer*-specific training annotation file. Augustus UTR prediction was disabled and only a single transcript was predicted for each putative gene locus.

In addition, gene predictions were also performed using Maker2 [110] with assembled Illumina-based transcriptome, PacBio IsoSeq transcriptome, and high quality proteins from Percomorphaceae taxon. Maker2 predicted 29,401 genes and 100,765 different proteins. For mapping PacBio IsoSeq to assemebled genome we used GMAP software [111].

#### Protein coding gene prediction—consensus dataset

The proteins predicted by Augustus and Maker2 were clustered with the proteome of the three-spined stickleback using OrthoMCL [112]. The resulting clusters were further analysed to verify that Augustus and Maker2 predictions originated from the same genomic locus. Based on these analyses, we classified the genes into four groups: 1) conserved predictions (Augustus and Maker2 proteins are orthologous, have a stickleback ortholog and originate from the same gene locus), 2) species specific predictions (Augustus and Maker2 proteins are orthologous and originate from the same gene locus) 3) unplaced conserved predictions (Augustus and Maker2 proteins are orthologous, have a stickleback ortholog but originate from different gene loci) and 4) uncertain predictions (the remaining predicted genes).

For the conserved and species-specific categories, the gene prediction (from Augustus or Maker2) that was the longer at the defined locus was chosen as the representative. Since manual verification had shown that Maker2 predictions were often truncated compared to Augustus, for unplaced conserved predictions, the gene at the locus identified by Augustus was chosen as the representative. Uncertain predictions were not included in the final consensus gene dataset. Transcripts in the consensus dataset were then given unique identifiers. The final dataset was filtered for potential duplicates.

### Functional annotation

The Asian seabass reference proteins were aligned to proteins annotated in SwissProt and TrEMBL databases [113,114] using blastp from NCBI BLAST package [115] with E-value set to 10-e5 and the best hit was chosen for each protein.

RunIprScan-1.1.0 client (http://michaelrthon.com/runiprscan/) was used for searching known protein motifs and domains by searching against publicly available databases available in InterPro [25], including Pfam, PRINTS, PROSITE, ProDom, and SMART. The Gene Ontology (GO) terms were retrieved from RunIprScan-1.1.0 results for each protein. The mapping to KEGG pathways [27] was computed using KAAS webserver [116]. The statistics for functional annotation are summarized in S26 Table.

### Protein clustering and alignment

We downloaded the proteomes of 19 species from Ensembl [28] and NCBI RefSeq [102]. These proteomes contained a total of 389,038 proteins and represented 13 ray-finned fishes (*Astyanax mexicanus*, *Cynoglossus semilaevis*, *Danio rerio*, *Dicentrarchus labrax*, *Gadus morhua*, *Gasterosteus aculeatus*, *Larimichthys crocea*, *Notothenia coriiceps*, *Oreochromis niloticus*, *Oryzias latipes*, *Takifugu rubripes*, *Tetraodon nigroviridis*, *Xiphophorus maculatus*), one cartilaginous fish (*Callorhincus milii*), one lobe-finned fish (*Latimeria chalumnae*), one reptile (*Anolis carolinensis*), one amphibian (*Xenopus tropicalis*), one bird (*Gallus gallus*) and human (*Homo sapiens*). These proteomes were clustered together with the *L*. *calcarifer* genome using blastp [81] and FastOrtho (http://enews.patricbrc.org/fastortho). All *vs* all BLAST (using blastp [81]) was performed to identify homologous proteins and these were clustered using FastOrtho and MCL [117] (FastOrtho is a reimplementation of the OrthoMCL [112] algorithm in C++ and allowed for fast clustering of proteins).

Protein clusters were aligned using MAFFT [118]. The alignments were filtered using a custom script to identify alignments with a low proportion of gaps. Gaps were defined as a column where greater than 40% of sequences in an alignment column was a gap character, and alignments where greater than 50% of the alignment consisting of gaps were considered to have a high proportion of gaps and these alignments were discarded. The remaining alignments were considered for potential gene duplication detection.

### Small RNA sequencing and analysis

Total RNA was extracted using the mirVana miRNA extraction kit (Life Technologies, Carlsbad, CA, USA) and further purified using the miRCURY RNA isolation kit (Exiqon, Vedbaek, Denmark) according to manufacturer’s instructions. RNA concentration and integrity was measured on the NanoDrop 8000 Spectrophotometer (ThermoFisherScientific, Waltham, MA, USA) and visually assessed by agarose gel electrophoresis with ethidium bromide staining. RNA was stored at -80°C. Small RNA libraries were constructed with HD adapters as previously described [119]. Briefly, 2 μg of total RNA was ligated to 3’ and 5’ HD adapters using commercially available enzymes and reagents. Ligated RNA products were reverse transcribed to cDNA and amplified by PCR. The cDNA products expected to contain 19–25 base pair inserts were selected and purified by 8% PAGE and ethanol precipitation. Libraries were sequenced on the HiSeq 2500 Ultra-High-Throughput Sequencing System (Illumina) at The Genome Analysis Centre (Norwich, United Kingdom).

FASTQ files were converted to FASTA format and HD adaptor sequences were trimmed by removing the first 4 bases of each read followed by the 3’ adaptor and preceding four bases. Sequences shorter than 18nt and comprised of two or fewer unique bases were removed from further analysis.

miRNAs were annotated by searching all animal precursor hairpins from miRBase [120] against the reference genome (E = 10e-6) generating a set of putative pre-miRNA sequences. Overlapping BLAST hits were merged and mature miRNAs annotated in miRBase [120] were searched against putative precursors using PatMaN [121], those hairpins with a match to a mature miRNA (with up to one mismatch) were then folded using RNAfold [122] and those forming a valid pre-miRNA hairpin structure were annotated as miRNAs. In order to further annotate miRBase [120] orthologues, we mapped our small RNA reads to the putative hairpins. Those with evidence of expression in our samples that were consistent with precise Dicer and Drosha processing were annotated as “high-confidence” miRNAs. Those with no evidence of expression or small RNA expression that was not consistent with precise miRNA biogenesis were classified as “low-confidence”.

New miRNAs were predicted using both miRCat [123] and miRDeep2 [124] using default parameters. Predictions were merged to obtain a non-redundant set of candidates and known miRNA families from miRBase [120] identified previously were removed from the predicted novel miRNA set. Small RNA reads were aligned to predicted miRNAs and the read alignment pattern and secondary structure were checked manually to ensure that they are consistent with canonical miRNA biogenesis. Any predictions that did not meet these criteria were removed.

### Detection of gene duplications

Best-fit models for each multiple protein sequence alignment were predicted using ProtTest 3.4 [125]. Phylogeny trees were generated from the multiple sequence alignments using PhyML 3.0 [126] with default parameters and the model selected by ProtTest, generating trees for 12,741 protein clusters.

A total of 2,439 alignments containing at least two Asian seabass sequences were identified using a customized python script. A best-fit evolutionary model was predicted for each multiple protein sequence alignment using ProtTest 3.4 prior to reconstructing a phylogenetic tree from each of these alignments using PhyML with 1000 bootstraps. A total of 2,190 phylogenetic trees were reconstructed and rooted using the elephant shark (*Callorhinchus milii*). Where no elephant shark was present in the cluster, the root was chosen by finding the midpoint of the tree using the `get_midpoint_outgroup’ method of the ETE2 software [127]. Asian seabass duplicate genes were identified by parsing the phylogenetic trees identified using ETE2's get_descendant_events implemented in a python script (show_duplicated_genes.py). Duplication events that yielded two neighbour leaf nodes containing *L*. *calcarifer* proteins were retained.

### Major Histocompatibility Complex class I (MHC-class I) genes

The locations of major histocompatibility complex class I (MHC-class I) genes in the Asian seabass genome were searched to determine the continuity of the Asian seabass genome assembly. The genome annotation dataset was mined for MHC-class I genes and their coordinates. A similar analysis was performed for the three-spined stickleback using a previously published list of stickleback MHC genes [128].

### GO term enrichment analysis

The list of duplicated proteins was filtered to exclude those proteins lacking GO annotation, yielding a list of 844 proteins. This set was analysed using the BiNGO plugin to Cytoscape [129]. BiNGO calculates a p-value from a Fisher Exact test that compares the prevalence of GO terms in the query set (genes that were duplicated: 844 proteins mapped to 458 GO terms) to the GO term prevalent in the proteome as a whole (16,671 proteins mapped to 2,984 GO terms). Two analyses were performed, to identify relatively over and under represented terms. The p-values were adjusted using the Benjamini-Hochberg correction. Terms with an adjusted p-value of less than 0.05 were considered significantly differentially expressed and were retained. Retained terms were visualised by mapping onto the GO ontology graph.

### Phylogenetic analyses

Previously reported one-to-one orthlogues [22] were used as a starting point to identify the corresponding orthologous sequences from *Cynoglossus semilaevis*, *Larimichthys crocea*, *Notothenia coriiceps* and *L*. *calcarifer* using RSD approach [130]. In total, we identified 313 strict one-to-one orthologues from the 24 species. Multiple alignments were generated using ClustalW version 2.0.12 [131]. Alignments were concatenated using an in-house perl script. Ambiguous regions of the alignment were removed using Gblocks version 0.91b [132]. We used RAxML version 8.1.3 [133] to generate a Maximum Likelihood (ML) tree. The best-fit substitution model for the alignment was deduced using a perl script (ProteinModelSelection.pl) available at the RAxML webpage [133]. The JTT+F model, as deduced by the script, was used for the ML analyses. Node support was estimated using 100 bootstrap replicates.

### B chromosomes

#### Fish samples and primary fibroblast cell culture

Fishes were obtained from our selection program based at the Marine Aquaculture Centre (MAC) of the Agri-Food and Veterinary Authority of Singapore (AVA), located on St John's Island, Singapore. Asian seabass larvae at the age of one to two days post-hatching (dph) were sacrificed on ice and used for culturing primary fibroblasts and for preparing chromosomes spreads as described previously [12].

#### Microdissection and amplification

Three separate B chromosomes from different chromosome spreads were microdissected and collected using a glass needle coupled with an inverted microscope into collection drop solution as described before [134]. After incubation at 60°C for an hour, the collection drop solution was transferred to 5μl of water. An initial round of B chromosome DNA amplification was performed using the WGA 1 Kit (Sigma-Aldrich). Primary PCR products were used for probe preparation (for FISH experiments) and amplified for further sequencing. The WGA-PCR-amplified chromosome material was re-amplified with 16-dUTP-biotin and digoxigenin-11-dUTP (both 2 μM, Roche) under the following conditions: (1×) 94°C for 5 min; (35×) 90°C for 30 s, 54°C for 30 s, 72°C for 30s using WGA3 re-amplification kit (Sigma).

#### Library construction, sequencing and assembly

Sequencing libraries were prepared using the NEBNext DNA Library Prep Master Mix Set for Illumina (Illumina) for the ChB6 and ChB5 libraries, and the Nextera DNA Sample Preparation Kit (Illumina) for the ChB1 library. Libraries were sequenced on the Illumina MiSeq System with read length configuration of 2х250 bp for the ChB6 and ChB5 libraries and 2x300 bp for the ChB1 library. In all, 343,987, 404,427 and 382,627 sequencing reads were generated for ChB1, ChB5 and ChB6, respectively.

All reads with quality score less than 20 bp were removed, adapter sequences (WGA-specific, TGTGTTGGGTGTGTTTGG) were trimmed using the Cutadapt program [135] and low quality bases were trimmed using “Trim Galore” (http://www.bioinformatics.babraham.ac.uk/projects/trim_galore/) with default parameters.

Clean reads were mapped to assembled reference Asian seabass genome using Bowtie2 [136] with default parameters. Successfully mapped reads were chained together across gaps less than 10 kb to form B chromosome pseudo-scaffolds. Pseudo-scaffolds were assembled using CAP3 [137] to remove redundancy with the following parameters: minimum 50 bp overlapping length and 85% of similarity. Contigs were manually checked to reduce potential mis-assemblies (S27 and S28 Tables).

#### Fluorescence In Situ Hybridization (FISH)

Tandem repeat probes were amplified from genomic DNA using the following primers: Lca_217 5’-GCCATTCTGAGCTGAATAAGCCTC-3’; Sat_LM 5’-CCAAAGAGAAGCACTTATGA-3’; and Lca_38 5’-Fc- AAAAAATGTCATAGTATAGTATGGCGTCAAAAAACATG-3’. The FISH procedure, slide preparation and image analysis were performed as described earlier[12]. Hybridization for precise B chromosomes identification was performed under high-stringency conditions [134]. Finally, the slides were counterstained with DAPI and mounted in an antifade solution (Vectashield from Vector laboratories, Burlingame, CA, USA).

Images were merged and measured using Image-Pro Express software V5.0 (Media Cybernetics, Rockville, MD, USA). Final image adjustments were performed using Adobe Photoshop CS2. The path of the chromosomes was computationally traced and straightened according to the manual provided by the Image J software V1.41 (http://rsb.info.nih.gov/ij).

### Morphometric analyses

The morphometric data was obtained from digital images of 65 individuals (22 from South-East Asia, 22 from India and 21 from Australia–Papua New Guinea) in a truss network system. The details of the methodology can be found in Ref#[8]. The statistic Box’s M which tests the hypothesis of equality of co-variances across groups was non-significant. Therefore, within-groups covariance matrix was used for estimating discriminant functions. Wilks’ lambda which is a measure of individual variable’s potential indicated that V18 variable is better at discriminating between groups compared to the other variables.

Only two variables (V18 and V3) contributed significantly to discrimination between groups and hence retained in the model (the remaining 16 variables were excluded from the analysis based on Wilks’ lambda criterion) and used for estimating standardized canonical discriminant function coefficients. The developed discriminant functions could correctly classify about 73.8% of the fish to the respective groups. The cross-validation procedure could correctly assign about 72.3% of fish to the respective groups. Overall, the discriminant function was able to correctly assign three out of every four fish to the respective groups. Principal component analysis was also performed based on the 18 truss measurements normalized by maximum length of fish using correlation matrix in Past 3.7 software [138].

### Analyses of Asian seabass populations by Whole Genome Resequencing (WGRS)

The details of 62 Asian seabass samples (the reference individual and 61 additional individuals used for re-sequencing) collected from 13 geographic regions across its range are given in S21 Table. Paired-end genomic DNA libraries were constructed and sequenced using the Illumina platform (S22 Table). The main steps for analyzing the sequence data are outlined in S7 Fig. Mapping quality of at least 40 and base quality of 17 were used for SNP calling. Using GATK unified genotyper [139], a total of 8,464,441 SNPs with Phred score > 30 were found in the 62 samples. Also, 6,522,041 SNPs with Phred score > 30 were found on repeating SNP-calling using Samtools pipeline [140]. The combined set of 6,458,484 SNPs common to both the SNP callers were filtered for repeat sequences using Tandem Repeat Finder [96] and Repeat Scout [141] (SNP calling in repeats is unreliable because of the high misalignment rate and problematic assembly of repeat sequences) resulting in 5,642,327 SNPs.

Principal component analysis (PCA) was performed using SNPrelate [142]. SNPs with less than 5% minor allele frequency and SNPs with linkage disequilibrium (LD) threshold more than 0.2 were removed from the analyses. The final set of results used for PCA analysis consisted of 64,634 SNPs (Figs 4 and S8).

Model-based inference of ancestry amongst the various seabass populations was performed using ADMIXTURE [143] software based on ML-optimization. All SNPs with more than 5% missing data were filtered out. ADMIXTURE model cannot incorporate loci in LD, so Plink 1.9 was used to remove SNPs with LD level more than 0.1 in 100 kb window. Final dataset for ADMIXTURE analyses consisted of 27,809 SNPs. The number of Ks which best explain the seen variation was also elucidated (Figs 5 and S9).

For population phylogenetic analyses, SNPs were filtered out with the following parameters: distance between SNPs of at least 4000 bp (to reduce the linkage effects), MAF = 0.05, maximum 3 missing genotypes per SNP. Final dataset comprised of 123,594 SNPs. Maximum-Likelihood tree was constructed using RaxMl 8 software [133] with GTR matrix and Gamma parameter using 100 bootstrap replicates. SNPs were annotated using SnpEff software [144].

### Sequence availability

The scaffolded genome assembly (v2) has been submitted to DDBJ/EMBL/NCBI GenBank under the accession LLXD00000000. Alternatively, it is also available for download at http://seabass.sanbi.ac.za/, together with the annotations (for the v2 assembly). The chromosome-level genome assembly (v3) is also available at the above-mentioned website. The Illumina and PacBio reads utilized for the genome assembly, as well as the whole-genome resequencing reads have been submitted to NCBI SRA under BioProject accession numbers SRP069219 and SRP069848, respectively. The BAC end sequences have been submitted to NCBI dbGSS under the accession numbers KS320706—KS326261 for the *Bam* HI library and KS326262—KS331896 for the *Eco* RI library.

## Supporting Information

S1 File Asian seabass tandem repeat consensus sequences. (DOCX)

S1 Fig Observed k-mer distribution and modeling results. **k-mer frequency counting analyses was done for the Illumina genomic reads.** Jellyfish [74] was used with the following parameters and commands: *jellyfish count -m 21 -s 100000000 -t 5 -o output -C InputFile* (counting 21-mer frequencies), *jellyfish merge -o output*.jf output_* (merging multiple output files), *jellyfish histo–h* 10000000 *-f output*.*jf > output_histogram*.*txt* (generating k-mer frequency histogram) and *jellyfish stats -v -o stats*.*txt output*.*jf* (generating statistics). Cov: Coverage.
(TIF)

S2 Fig The number of contigs in the primary Asian seabass genome assembly (v1; 3,917 contigs) compared to those of published fish genome assemblies (see S23 Table for more details). (TIF)

S3 Fig **Evaluation of the Asian seabass scaffolded genome assembly (v2) by mapping Illumina PE Genome reads to assembly for linear insert size libraries in the size range of 500 bp (A) and 750 bp (B).** The 80X Illumina paired-end HiSeq genome sequence data was mapped to the PacBio-based assembled genome using the CLC Genomics Workbench version 8.5.1 mapping tool. The following parameters were applied: (i) alignment similarity cut-off at 90% and (ii) at least 90% of the read must match the reference sequence. CLCbio's autodetect feature was used to determine the paired distance range. For the 500 bp library (A), the estimated paired distance range was 380 to 580 bp while for the 750 bp library (B), the estimated paired distance range was 580 to 780 bp.
(TIF)

S4 Fig A screenshot of the Asian seabass genome assembly (v1) showing a location wherein a ~15 kb region missed by short reads has been captured using long reads from PacBio sequencing. (TIF)

S5 Fig **Comparison of GC content of Asian seabass genome assembly (v2)with few selected fish genomes (A), with representatives from the different classes of vertebrates (B) and comparison of GC content with genome size of selected fishes (C).** The GC-content of genomes of interest were calculated using a 20 kb sliding window (BedTools utilities [145]). In addition to *Lates calcarifer*, the genomes analyzed included (A) six teleosts (*Danio rerio*, *Gadus morhua*, *Gasterosteus aculeatus*, *Oryzias latipes*, *Takifugu rubripes*, *Tetraodon nigroviridis*) or (B) six vertebrates (*Anolis carolinensis*, *Callorhinchus milii*, *Gallus gallus*, *Homo sapiens*, *Petromyzon marinus*, and *Xenopus tropicalis)*. Sliding windows with more than 25% of Ns (gaps) were discarded and the proportion of sliding windows with a given GC-content (%) was calculated and plotted. The script utilized to run BedTools [145] and perform downstream processing is available at https://github.com/ramadatta/Scripts/blob/master/Average_GC_Content_Analysis/knowGC-contentrun1.sh. (C) Genome size of selected fish genomes compared with their average GC content. BP: *Boleophthalmus pectinirostris*; DR: *Danio rerio*; GM: *Gadus morhua*; GA: *Gasterosteus aculeatus*; LC: *Lates calcarifer* NB: *Neolamprologus brichardi*; OL: *Oryzias latipes*; ON: *Oreochromis niloticus*; TR: *Takifugu rubripes*; TN: *Tetraodon nigroviridis*.
(TIF)

S6 Fig Functional annotation of Asian seabass protein-coding genes. The number of genes in top ten entries for A) Interpro B) KEGG pathways and C) GO.
(TIF)

S7 Fig Pipeline for Asian seabass population data analyses. The flowchart outlines the steps taken for analyses of Asian seabass genome sequence information from 62 fishes collected from 13 regions across its geographic range.
(TIF)

S8 Fig PCA plots of Asian seabass populations using SNPs. **A total of 64,634 SNPs were used for PCA analyses.** The results at different percentages of explained variation are shown in A) and B).
(TIF)

S9 Fig Cross-validation error analyses to identify the number of Ks which explain variation in the Asian seabass species complex. Cross-validation methodology was used to find number of Ks (clusters/population) which better explain observed variation. The best model was obtained at K = 3, with the lowest error level.
(TIF)

S10 Fig Maximum likelihood (ML) tree constructed using 123,594 SNPs from *Lates calcarifer* with Indian region (red), S-E Asia/Philippines (green) and Australia/Papua New Guinea (blue). (TIF)

S11 Fig The Asian seabass genome assembly (v2; blue bars) anchored to the 24 linkage groups (white bars) using 772 markers [21]. Regions indicated in red represent positions of contig/scaffold containing Lca_217 (peri-centromeric sequences).
(TIF)

S12 Fig Truss morphometric analyses of Asian seabass individuals collected from three regions. Purple and green lines represent truss measurements with blue circles indicating the landmark regions. The descriptions for the landmarks are, 1—tip of the snout, 2—point on dorsal surface of fish that is exactly perpendicular to the base of pectoral fin, 3—anterior base of dorsal fin, 4—posterior base of dorsal fin, 5—dorsal base of caudal fin, 6—base of central caudal fin rays, 7—ventral base of caudal fin, 8—posterior base of anal fin, 9—anterior base of anal fin and 10—base of pelvic fin (A & B). The landmark 6 is utilized only for generating the standard length of the fish. The remaining 9 landmarks were used to generate 18 inter-landmark truss measurements as indicated in panel A. The variables V18 and V3 (indicated with green colour in panel B) were observed to be more important for discriminating the three groups of fishes than other variables based on Wilks’ lambda criterion, coefficients of discriminant function and coefficients of structure matrix. Representative fishes from Australia-Papua New Guinea (C&D), SE Asia (E&F) and Indian region (G&H) are shown.
(TIF)

S13 Fig Map of the tropical Asia Pacific region showing the sampling locations for Asian seabass across its native range. India-Western coast (orange), India-Eastern coast (brown), Cambodia (red), Thailand-Eastern Coast (purple), Vietnam (pink), Singapore (black), Philippines (yellow), Indonesia-South Jakarta (green), Indonesia-Kalimantan (dark green), Indonesia-Sulawesi (white), Papua New Guinea (grey), Australia-Darwin (blue) and Australia-Queensland (light blue).
(TIF)

S14 Fig The Asian seabass genome assembly contains a more continuous cluster of MHC-class I genes compared to the well-assembled *G*. *aculeatus* genome. The *L*. *calcarifer* MHC-class I genes were found to be located on eight contigs/scaffolds, four of which were placed onto linkage group 3 (LG3). Four of these eight contigs/scaffolds were also >1Mb in length. The dashed connecting-lines indicate gaps introduced during sequence placement of contigs/scaffolds into linkage groups, while the yellow bars within the “scaffold_” sequences indicate Ns introduced during scaffolding. To allow for comparison at the level of contigs/scaffolds, the *G*. *aculeatus* chromosome groupX was split at the gapped regions (indicated by the dashed connecting-lines). The *G*. *aculeatus* MHC-class I genes were found to occupy 14 contigs/scaffolds, all except one being <113 kb in length.
(TIF)

S1 Table Metrics of the Asian seabass genome assemblies. (XLSX)

S2 Table RepeatMasker output file tabulating the masking results for vertebrate repeat sequences (A)* and for Asian seabass-specific repeat sequences (B)^.
(XLSX)

S3 Table Percentage of repeats in the Asian seabass genome obtained by various tools. (XLSX)

S4 Table Details of microsatellites identified in the Asian seabass genome assembly (v2). (XLSX)

S5 Table Tandem repeats with highest coverage of 23-mer HiSeq reads. (XLSX)

S6 Table Asian seabass repeat libraries. (XLSX)

S7 Table Summary of transposable elements identified in the Asian seabass genome. (XLSX)

S8 Table Statistics of tRNAs identified in the Asian seabass genome assembly. (XLSX)

S9 Table *In silico* enzyme selection for optical mapping. (XLSX)

S10 Table Three evaluated MapCards for optical mapping. (XLSX)

S11 Table Whole genome MapCard collection summary for optical mapping. (XLSX)

S12 Table *De novo* assembly of Single Molecule Restriction Maps. (XLSX)

S13 Table Statistics of sequence placement (only sequences ≥40kb) on assembled Maptigs. (XLSX)

S14 Table Placement of genome sequences ≥40kb on assembled Maptigs. (XLSX)

S15 Table Inventory of potentially misassembled sequences identified by the optical map data. (XLSX)

S16 Table Chromosome-level assembly of the Asian seabass genome (v3). (XLSX)

S17 Table Inventory of 247 overlaps between ends of neighbouring contigs that were closed during scaffolding of the Asian seabass genome. (XLSX)

S18 Table Summary statistics of synteny analyses. (XLSX)

S19 Table Summary of synteny blocks shared between *L*. *calcarifer* and *D*. *labrax*. (XLSX)

S20 Table Summary of synteny blocks shared between *L*. *calcarifer* and *G*. *aculeatus*. (XLSX)

S21 Table Sample collection details for Asian seabass whole genome resequencing effort. (XLSX)

S22 Table Details of Asian seabass whole genome resequencing effort. (XLSX)

S23 Table Summary of sequenced fish genomes. (XLSX)

S24 Table Truss morphometric analyses of Asian seabass individuals from the three regions. (XLSX)

S25 Table Comparison of annotation statistics across a few fish genomes. (XLSX)

S26 Table Functional classification of the Asian seabass gene set. (XLSX)

S27 Table Read statistics for B chromosome-derived sequences. (XLSX)

S28 Table Assembly of B chromosome-derived fragment statistics. (XLSX)

S29 Table Comparison of SNP distribution across the exons, introns, intergenic and UTR regions. (XLSX)

S30 Table Functions over-represented in duplicated genes of the Asian seabass. (XLSX)

S31 Table Functions depleted in duplicated genes of the Asian seabass. (XLSX)
