# Correction: Chromosomal-Level Assembly of the Asian Seabass Genome Using Long Sequence Reads and Multi-layered Scaffolding

**DOI:** 10.1371/journal.pgen.1006500

**Published:** 2016-12-09

**Authors:** Shubha Vij, Heiner Kuhl, Inna S. Kuznetsova, Aleksey Komissarov, Andrey A. Yurchenko, Peter Van Heusden, Siddharth Singh, Natascha M. Thevasagayam, Sai Rama Sridatta Prakki, Kathiresan Purushothaman, Jolly M. Saju, Junhui Jiang, Stanley Kimbung Mbandi, Mario Jonas, Amy Hin Yan Tong, Sarah Mwangi, Doreen Lau, Si Yan Ngoh, Woei Chang Liew, Xueyan Shen, Lawrence S. Hon, James P. Drake, Matthew Boitano, Richard Hall, Chen-Shan Chin, Ramkumar Lachumanan, Jonas Korlach, Vladimir Trifonov, Marsel Kabilov, Alexey Tupikin, Darrell Green, Simon Moxon, Tyler Garvin, Fritz J. Sedlazeck, Gregory W. Vurture, Gopikrishna Gopalapillai, Vinaya Kumar Katneni, Tansyn H. Noble, Vinod Scaria, Sridhar Sivasubbu, Dean R. Jerry, Stephen J. O'Brien, Michael C. Schatz, Tamás Dalmay, Stephen W. Turner, Si Lok, Alan Christoffels, László Orbán

There is an error in the third paragraph of the Introduction. Specifically, the following sentence is incorrect: "The karyotype is represented by a diploid number of A chromosomes (2n = 24) and a variable number (2–10) of additional B chromosomes [12]". This sentence should read: "The karyotype is represented by a diploid number of A chromosomes (2n = 48) and a variable number (2–10) of additional B chromosomes [12]".
