# Supplementary material for: Chromosomal-Level Assembly of the Asian Seabass Genome Using Long Sequence Reads and Multi-layered Scaffolding
Source: PLoS Genet. 2016 Apr 15;12(4):e1005954. doi: 10.1371/journal.pgen.1005954 (PMC4833346; doi:10.1371/journal.pgen.1005954)
Supplement: S1 File — (DOCX) [file pgen.1005954.s001.docx]

**S1 File.** Asian seabass tandem repeat consensus sequences.

>Asian seabass ONSAT_SB Concensus_Lenght_170_bp

CGAAAAATTTAATAATTTAGGGGTCTTGAGCATGGGCGTGGTAAAATGCCCTCGGTAGCGCCACCTACATTTTTAAACGGAACAGCCCCTCAAGCCCGTTGCGCCTAAAAATCTGAAAATCTGCACACATATGTAACATCCCATGACGCACCAAAAAGTCTCTTGGAGCCA

>Asian seabass MOSAT_SB, T_34a, satellite DNA

TTTGTGACATCACTACATAGTTTGTTGAAAACGTAC

>Asian seabass MOSAT_SB, T_34b, satellite DNA

TTTGTGACATCACACATAGTTGTGGGTCAGTAC

>Sat_38

AAAAAATGTCATAGTATAGTATGGCGTCAAAAAACATG

>Sat_217 Consensus_Length_217_bp

AGTAAACAAGCATTATGGTTGAAACCATAATTTCCTGTCGGGAGAGCCTTTCCCTCTTTTGTGCACTGTATGCAATCCCAGAGTGTGAATAAGCGCTTTTCCAGCGTTTTGAGGCTTATTCAGCTCAGAATGGCTTAATACTGCACTATCTGACCAGGACAAAGACAAACTAAGAACTCAGCCACACGGACATGAAAGTTGTTTTACTTACAATATT

>Sat_217 Consensus_Length_427_bp

CCAGCGTTTTGAGGCTTATTCAGCTCAGAATGGCTTAATACTGCACTATCTGACCAGGACAAGACAAACTAAGAACTCAACACACTGACATGAAAGTTTCTATATTTCTATTAAAATAAACAAACATTATGGTTGAAACCATAATTTCATTTCGGGAGAGCCTTTCCCTCTTTGTGGCACTGTATGTAATCTGAAGTGTGAATAACGCTTTTCCCGCGTTTGAGGCTTATTCAGCTCAGAATGGCTTAATACTGCACTATCTGACCAGGACAAGACAAACTAAGAACTCAGCCACACGGACATGAAAGTTGTTTACTTTACAATATTAGTAAACAAGCATTATGGTTGAAACCATAATTTCCTGTCGGGAAGAGCTTCCCTCTTTTTGTGCACTGTATGCAATCCCAGAGTGTGAATAAGCGCTTTT

>Sat_LM_Consensus_Length_453_bp

CCCAACAAAATAGCACTTACTGCTTTCCAAATTCAAGTCTGTGCCTGTGTTAGGGTAATGTTGGTGATAAATTGATGTACTTAGCTTATCCTGTGAAAATGGTGTAAAATGGCAGTCTCCATGTTTGTTGAAGAAACTCACAACCAAGACAACCCCAAAGAGAAGCACTTATGAAGACAGAAATCATGAGTGGCTCTTATTGAAGTGATCAGAGTTGGACTCCATTAAGAAAATGGCTTTCAATGTAGTCAGTTGCTTTGTCACTTAAAACTGCCCCAACAAGTAGCACTTACTGCAATTTGGTAGTCAAAGTCAATGCTCAAGTTAAGGGTAATAGTAGTGACATAATAGAAAAATCTCTAAATATATTTGCTGGCAGCTTTAATATACAGAATGAGTGCCATGATGAATTCTTGATACAGAACAGGGACTTCCAAAATCAGCCAACACTAA

>Sat_LM_Consensus_Length_218_bp

CCCAACAAAATAGCACTTACTGCTTTCCAAATTCAAGTCTGTGCCTGTGTTAGGGTAATGTTGGTGATAAATTGATGTACTTAGCTTATCCTGTGAAAATGGTGTAAAATGGCAGTCTCCTGTTTGTTGAAGAAACTCACAACCAAGACAACCCCAAAGAGAAGCACTTATGAAGACAGAAATCATGAGTGGCTCTTATTGAAGTGATCAGAGTTGG

>Sat_LM Consensus Length_150_bp

CTCACAACCAAGACAACCCCAAAGAGAAGCACTTATGAAGACAGAAATCATGAGTGGCTCTTATTGAAGTGATCAGAGTTGGACTCCATTAAGAAAATGGCTTTCAATGTAGTCAGTTGCTTTGTCACTTAAAACTGCCCCAACAAGTAGCA

>T_261 CTGTCTCGGCTTCTGCTCGCTCATTAAGCTCAGAATTATCAAATGCTGTCTCCAGTGAACGGAGAAGAGAAGATGAGATTTCAGCCACTTTACTATGAATATTGGTCCAATTCTAATTTTCATGAACTGTAGGGATGGATGGAAACAGTCCGTGACCGAGGGGAAACATTTTCCCGTCTGTGAACTCACAGTGAGTCCCGCGTCTGAAAGTCTTGCTGTCTCGGATTCTGCTCACGGTGATTCCCGCGTCTGAAAGTCCTC

>T_213a

CACTAGACAGCAGTTTTTCATTCTGAGCTTAGTGAGCGAGCAGAAGCCGAGACAGGAGGACTTTCAGACGCGGGACTCAATGTGAGTCGACAGACGGGAAAAAGATCCCCCTCGGCGGCTCACACTTTCCATACATCCCGACTGTACATGATCAATAGAATTTGACCAATATTCATATAAAAGTGAATGAGATTTTACTTTCCCCTCTCCTTT

>T_213b

TTAAAAGGAGAGTGGAAATCTAAATCTCAGCCTCTCTAACACGAATCTATGTCAATTTGTCATGTTTACATTCAGAATGTCTCAAAGCAATCATAGCCGAGGGGAAACCTGTTGTCTGCTGTGGGTGCTCTAAGTGAGTCGAGTGTGTGAACATGCACCTTACGCAGCATTTGGACGCTAAATAAGCTCATAATGAACAACTGCTGTACCCTG

>T­_47

GGCCCCTTGCCCTAACCCTAACCTGCTTGGGCCCCTTGCCTAACCCTAACCTGCG

>T_80 CAGAGACTAAGTGTGAGCACAGGGAGAAGGGGAGATTTCCCCTTCTTCCCTTCCGAGCGATAGTATGCTCCCCTTCTCCCCTGTGCTCTCTC

>T_33a

ATTCAGCGTAATGTTTGTAAACATTCAGCATAATGTTTGTAAAC

>T_33b

ATTTGGCTGAATGTTTACAAACATTACGCTGAATGTTTACAAACATTA
