# Supplementary figures and images for: Chromosomal-Level Assembly of the Asian Seabass Genome Using Long Sequence Reads and Multi-layered Scaffolding
Source: PLoS Genet. 2016 Apr 15;12(4):e1005954. doi: 10.1371/journal.pgen.1005954 (PMC4833346; doi:10.1371/journal.pgen.1005954)

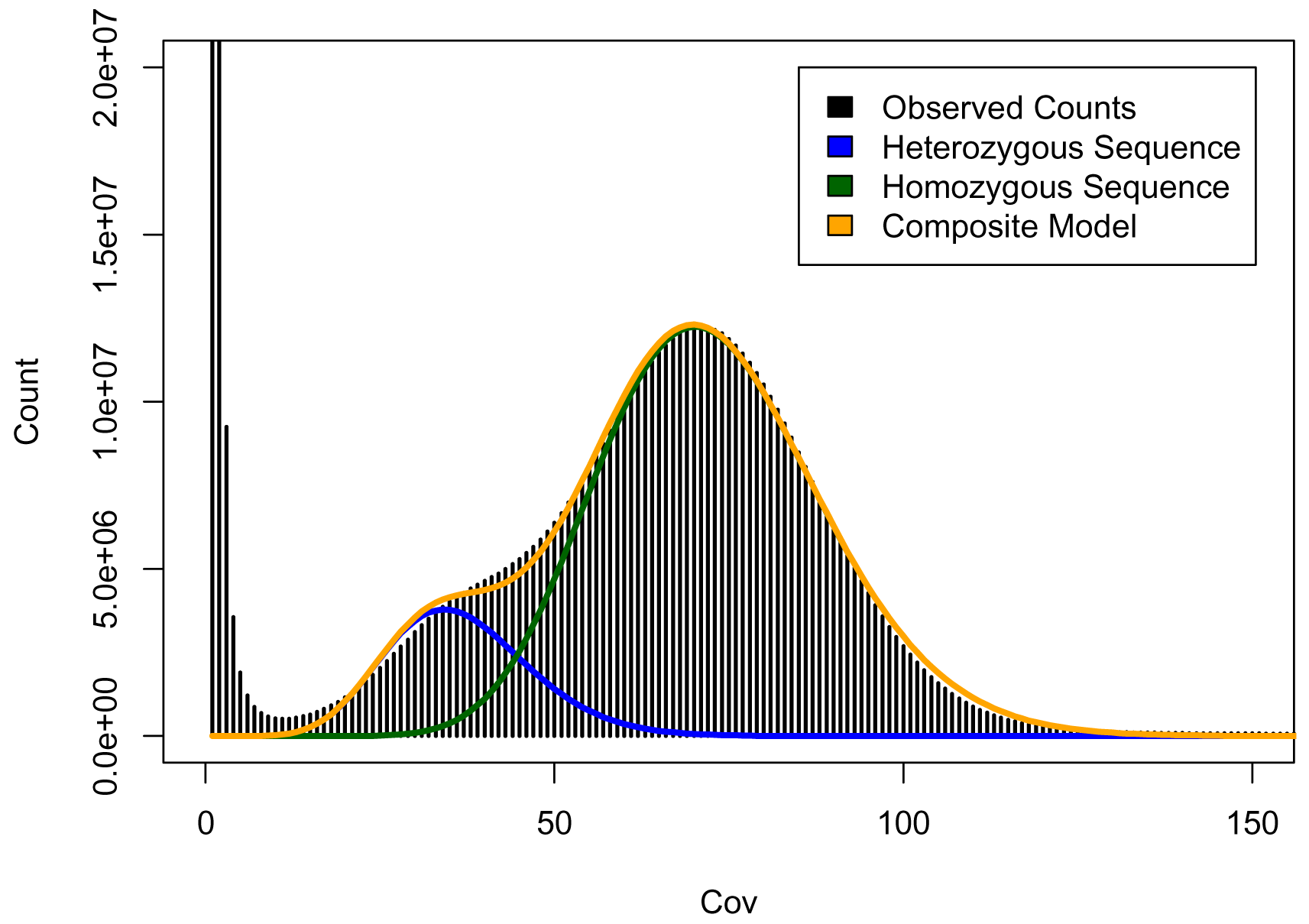

Supplement: S1 Fig — k-mer frequency counting analyses was done for the Illumina genomic reads. Jellyfish [74] was used with the following parameters and commands: jellyfish count -m 21 -s 100000000 -t 5 -o output -C InputFile (counting 21-mer frequencies), jellyfish merge -o output.jf output_* (merging multiple output files), jellyfish histo–h 10000000 -f output.jf > output_histogram.txt (generating k-mer frequency histogram) and jellyfish stats -v -o stats.txt output.jf (generating statistics). Cov: Coverage. (TIF) [file pgen.1005954.s002.tif]

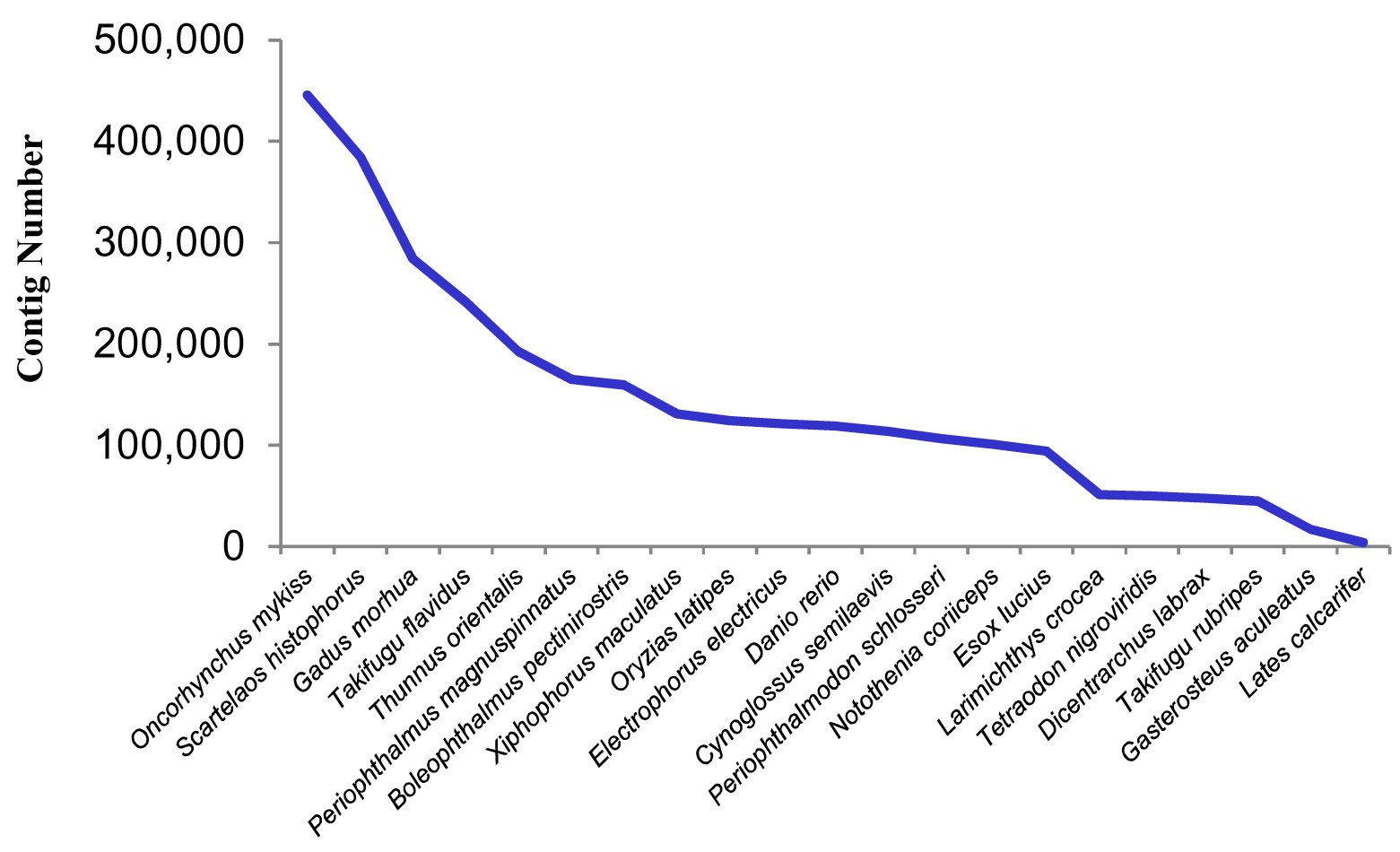

Supplement: S2 Fig — (TIF) [file pgen.1005954.s003.tif]

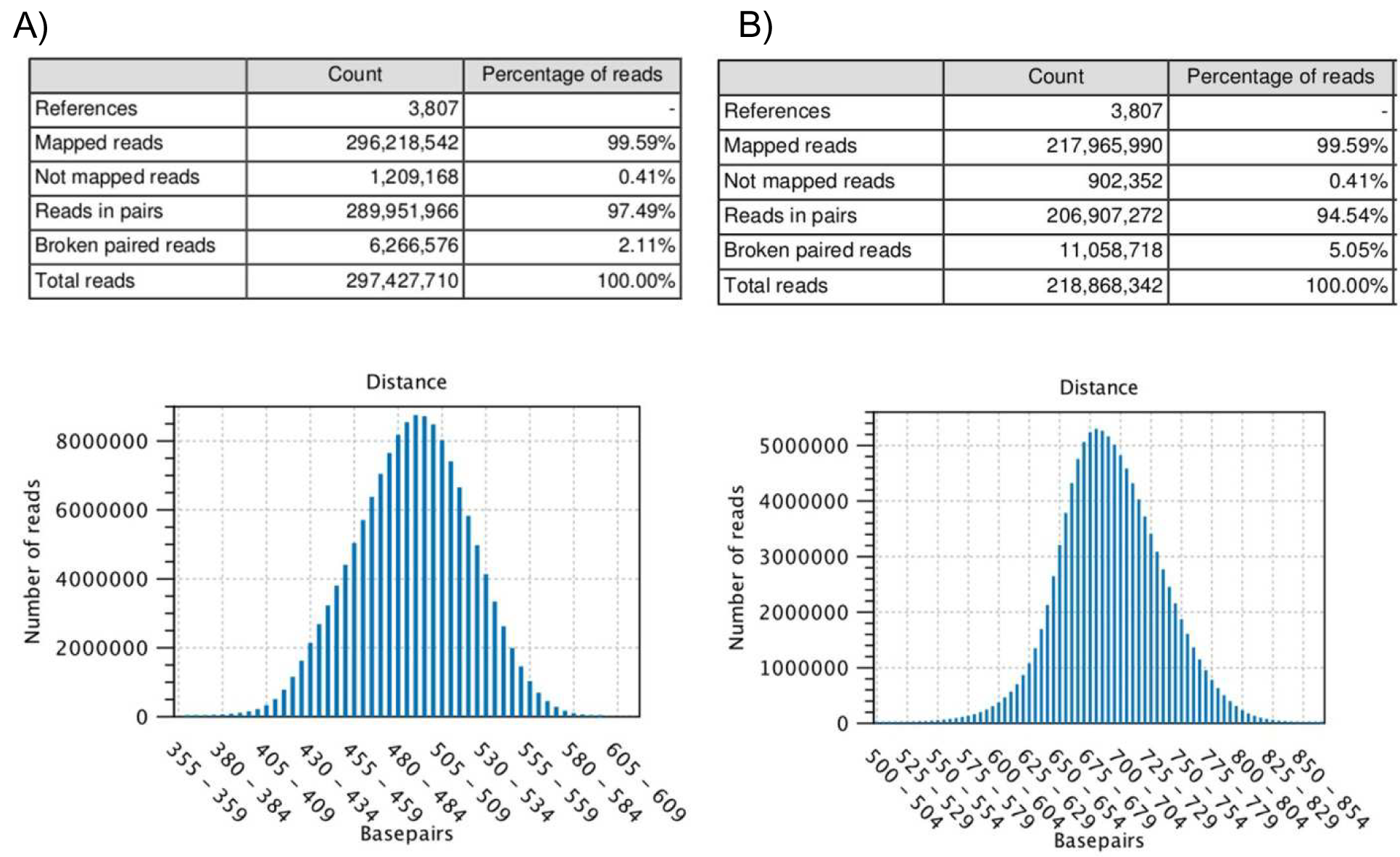

Supplement: S3 Fig — Evaluation of the Asian seabass scaffolded genome assembly (v2) by mapping Illumina PE Genome reads to assembly for linear insert size libraries in the size range of 500 bp (A) and 750 bp (B). The 80X Illumina paired-end HiSeq genome sequence data was mapped to the PacBio-based assembled genome using the CLC Genomics Workbench version 8.5.1 mapping tool. The following parameters were applied: (i) alignment similarity cut-off at 90% and (ii) at least 90% of the read must match the reference sequence. CLCbio's autodetect feature was used to determine the paired distance range. For the 500 bp library (A), the estimated paired distance range was 380 to 580 bp while for the 750 bp library (B), the estimated paired distance range was 580 to 780 bp. (TIF) [file pgen.1005954.s004.tif]

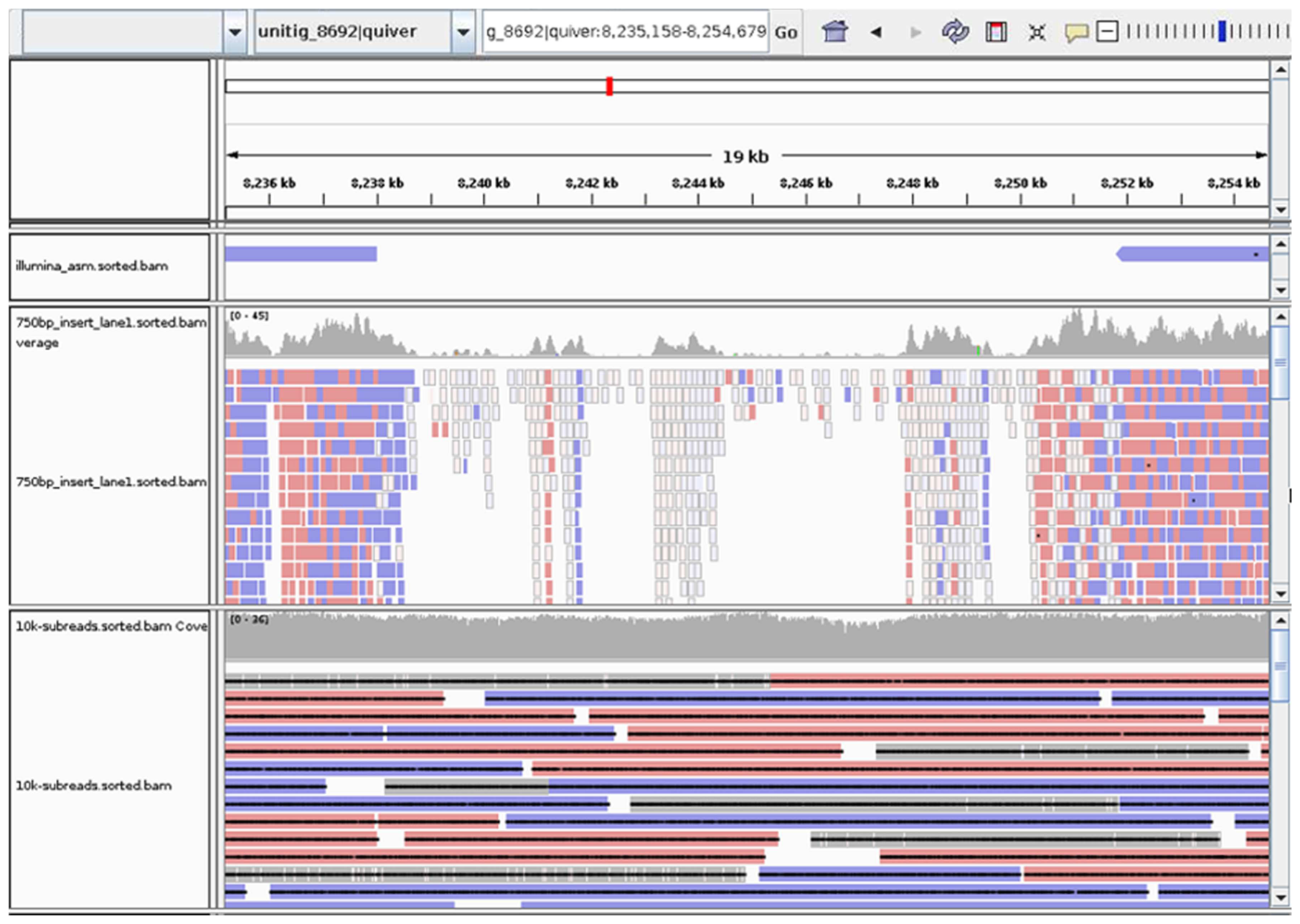

Supplement: S4 Fig — (TIF) [file pgen.1005954.s005.tif]

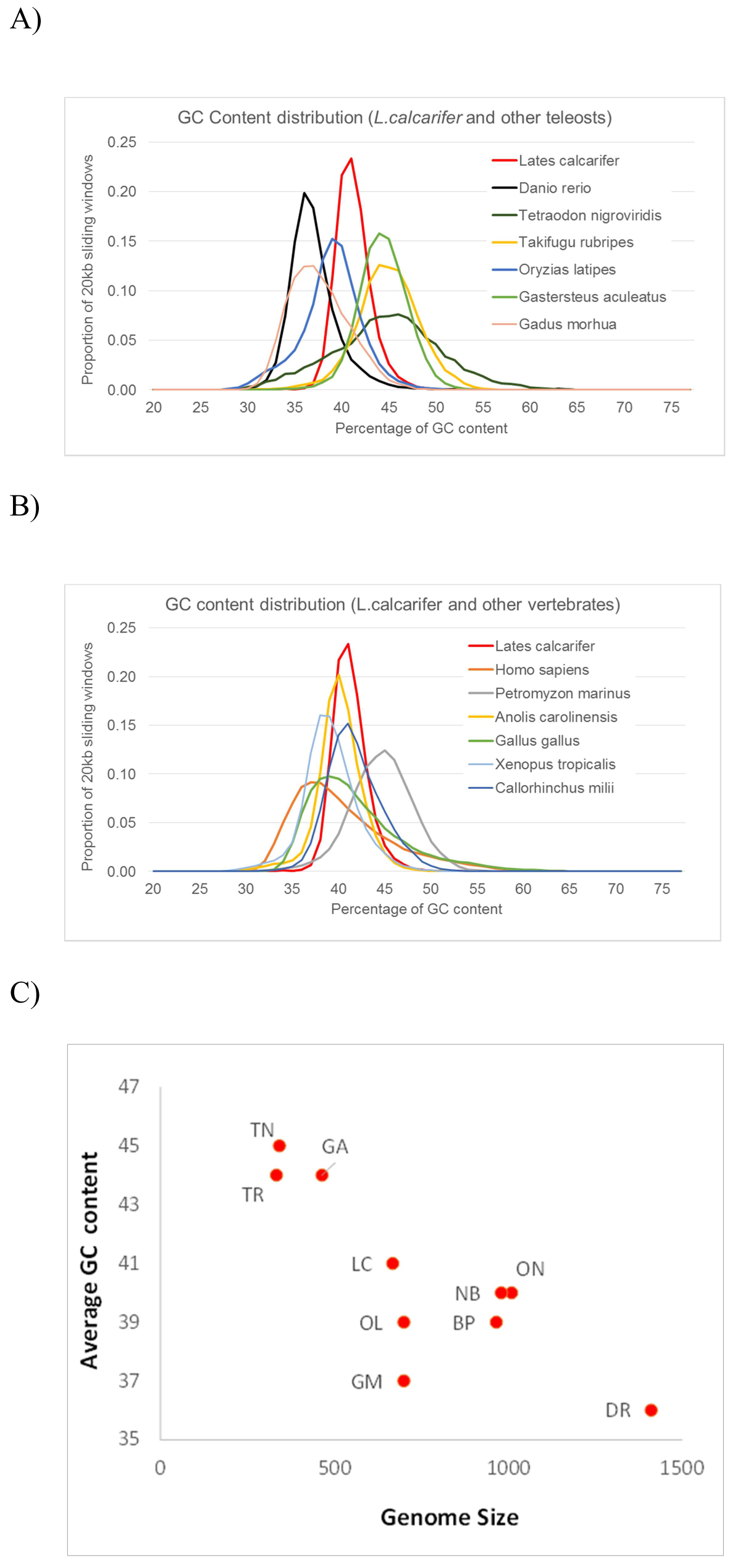

Supplement: S5 Fig — Comparison of GC content of Asian seabass genome assembly (v2)with few selected fish genomes (A), with representatives from the different classes of vertebrates (B) and comparison of GC content with genome size of selected fishes (C). The GC-content of genomes of interest were calculated using a 20 kb sliding window (BedTools utilities [145]). In addition to Lates calcarifer, the genomes analyzed included (A) six teleosts (Danio rerio, Gadus morhua, Gasterosteus aculeatus, Oryzias latipes, Takifugu rubripes, Tetraodon nigroviridis) or (B) six vertebrates (Anolis carolinensis, Callorhinchus milii, Gallus gallus, Homo sapiens, Petromyzon marinus, and Xenopus tropicalis). Sliding windows with more than 25% of Ns (gaps) were discarded and the proportion of sliding windows with a given GC-content (%) was calculated and plotted. The script utilized to run BedTools [145] and perform downstream processing is available at https://github.com/ramadatta/Scripts/blob/master/Average_GC_Content_Analysis/knowGC-contentrun1.sh. (C) Genome size of selected fish genomes compared with their average GC content. BP: Boleophthalmus pectinirostris; DR: Danio rerio; GM: Gadus morhua; GA: Gasterosteus aculeatus; LC: Lates calcarifer NB: Neolamprologus brichardi; OL: Oryzias latipes; ON: Oreochromis niloticus; TR: Takifugu rubripes; TN: Tetraodon nigroviridis. (TIF) [file pgen.1005954.s006.tif]

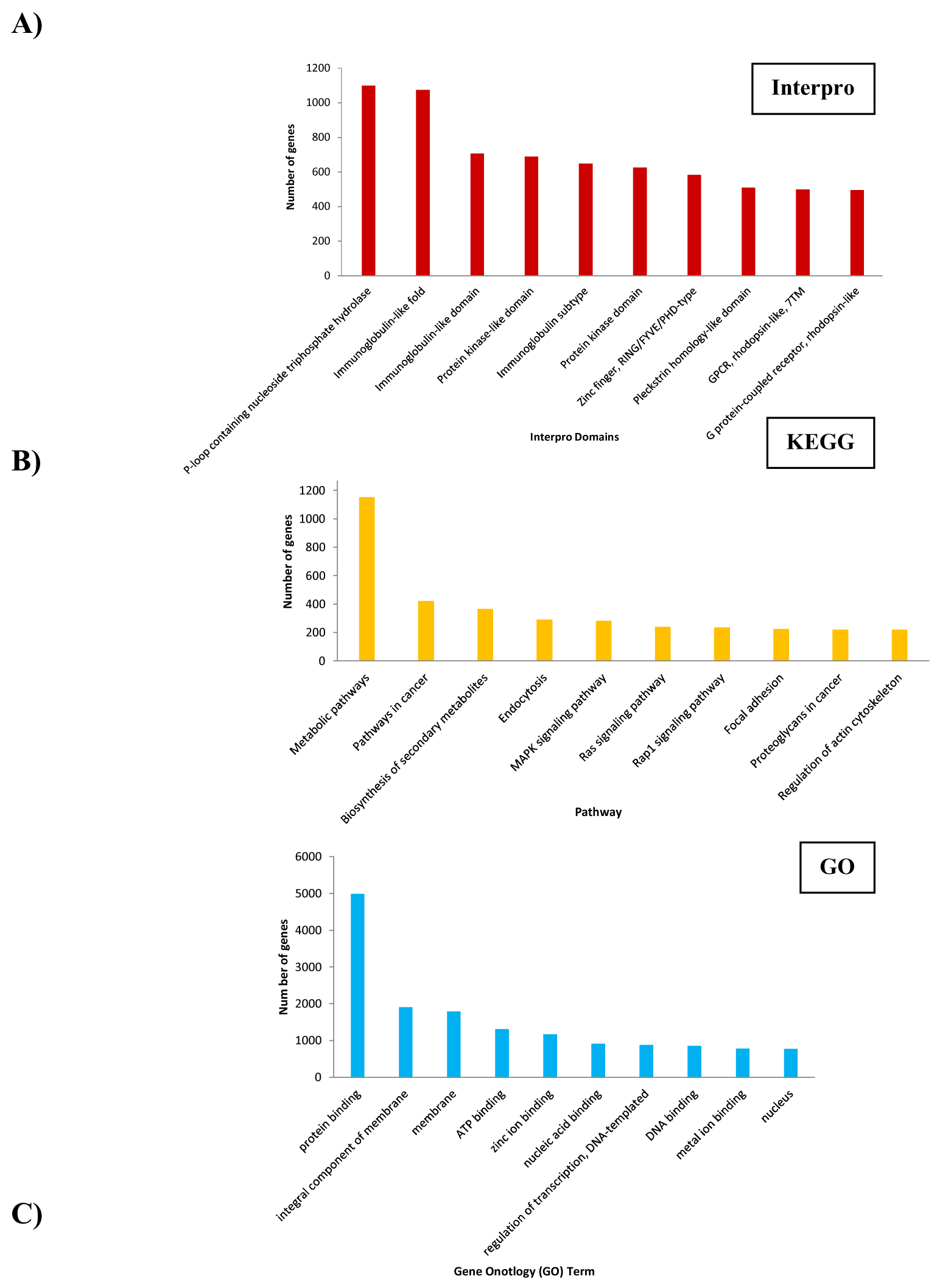

Supplement: S6 Fig — The number of genes in top ten entries for A) Interpro B) KEGG pathways and C) GO. (TIF) [file pgen.1005954.s007.tif]

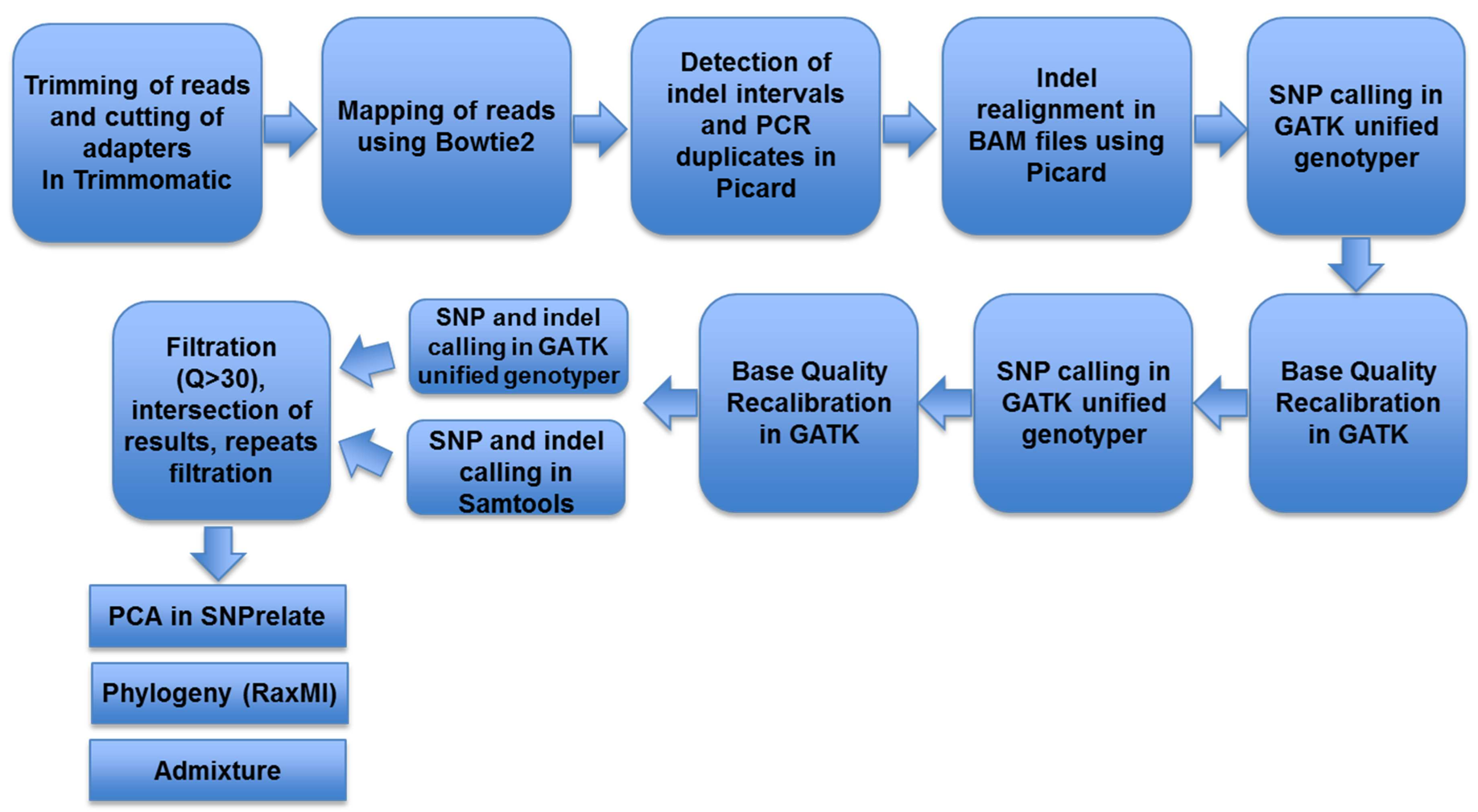

Supplement: S7 Fig — The flowchart outlines the steps taken for analyses of Asian seabass genome sequence information from 62 fishes collected from 13 regions across its geographic range. (TIF) [file pgen.1005954.s008.tif]

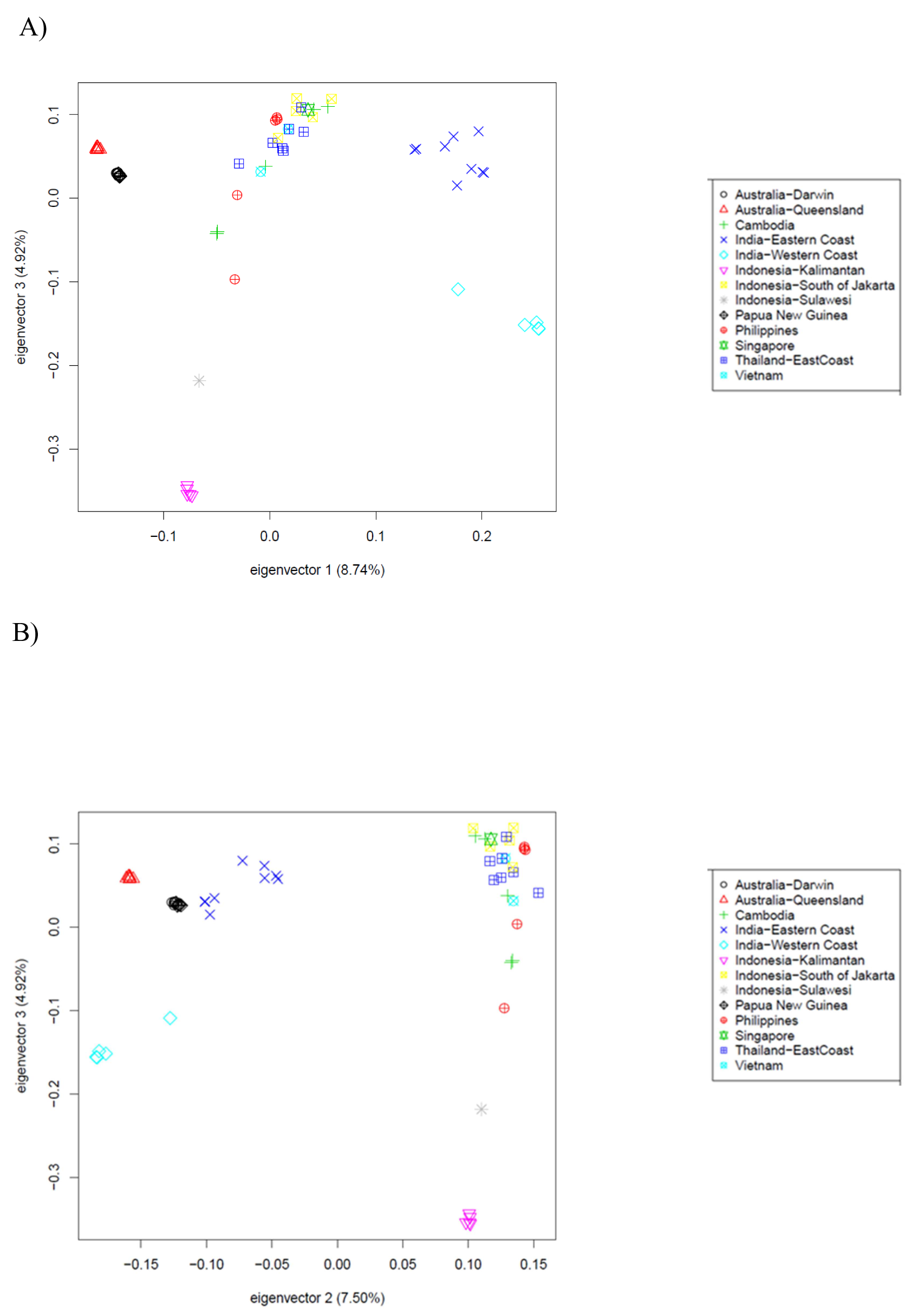

Supplement: S8 Fig — A total of 64,634 SNPs were used for PCA analyses. The results at different percentages of explained variation are shown in A) and B). (TIF) [file pgen.1005954.s009.tif]

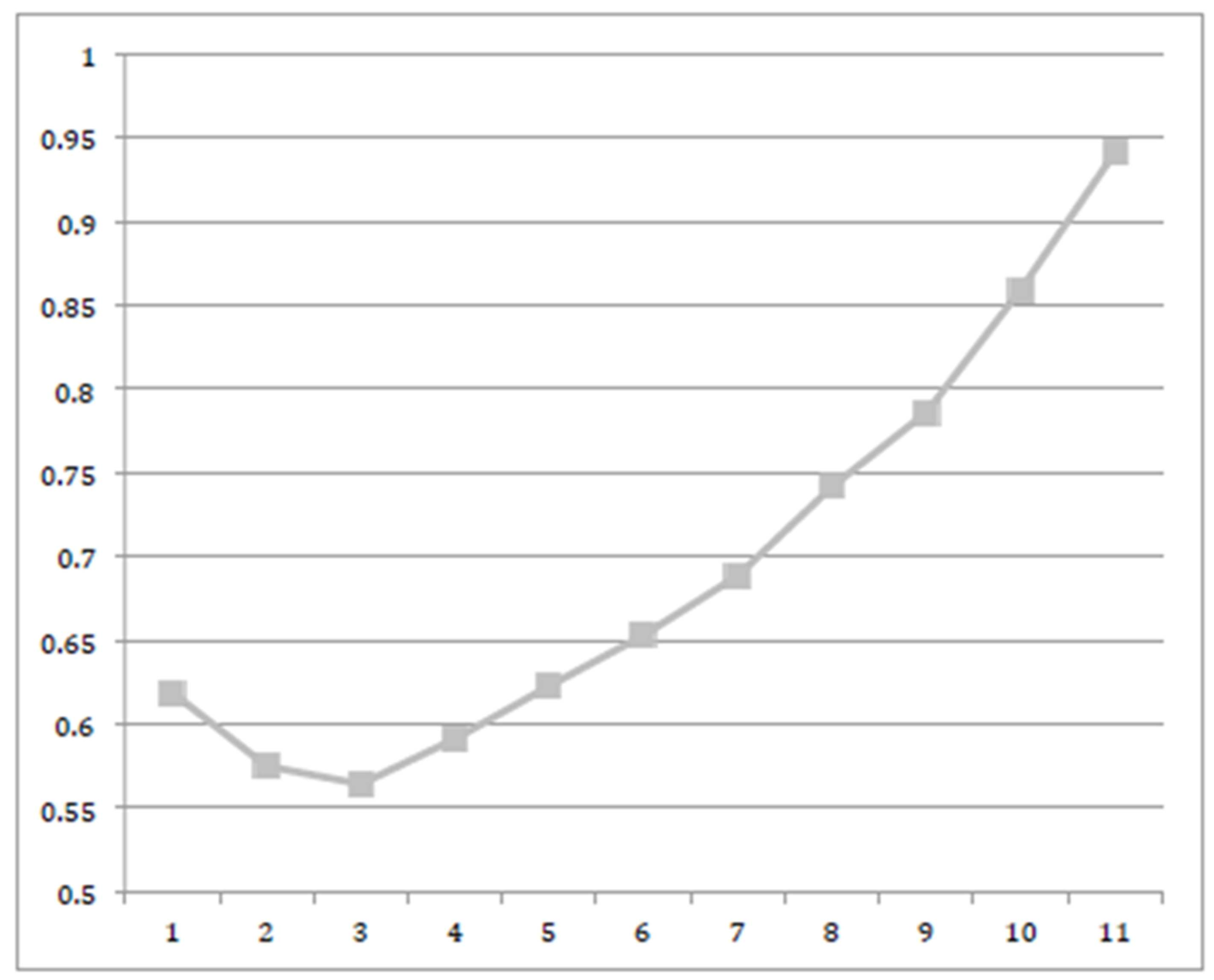

Supplement: S9 Fig — Cross-validation methodology was used to find number of Ks (clusters/population) which better explain observed variation. The best model was obtained at K = 3, with the lowest error level. (TIF) [file pgen.1005954.s010.tif]

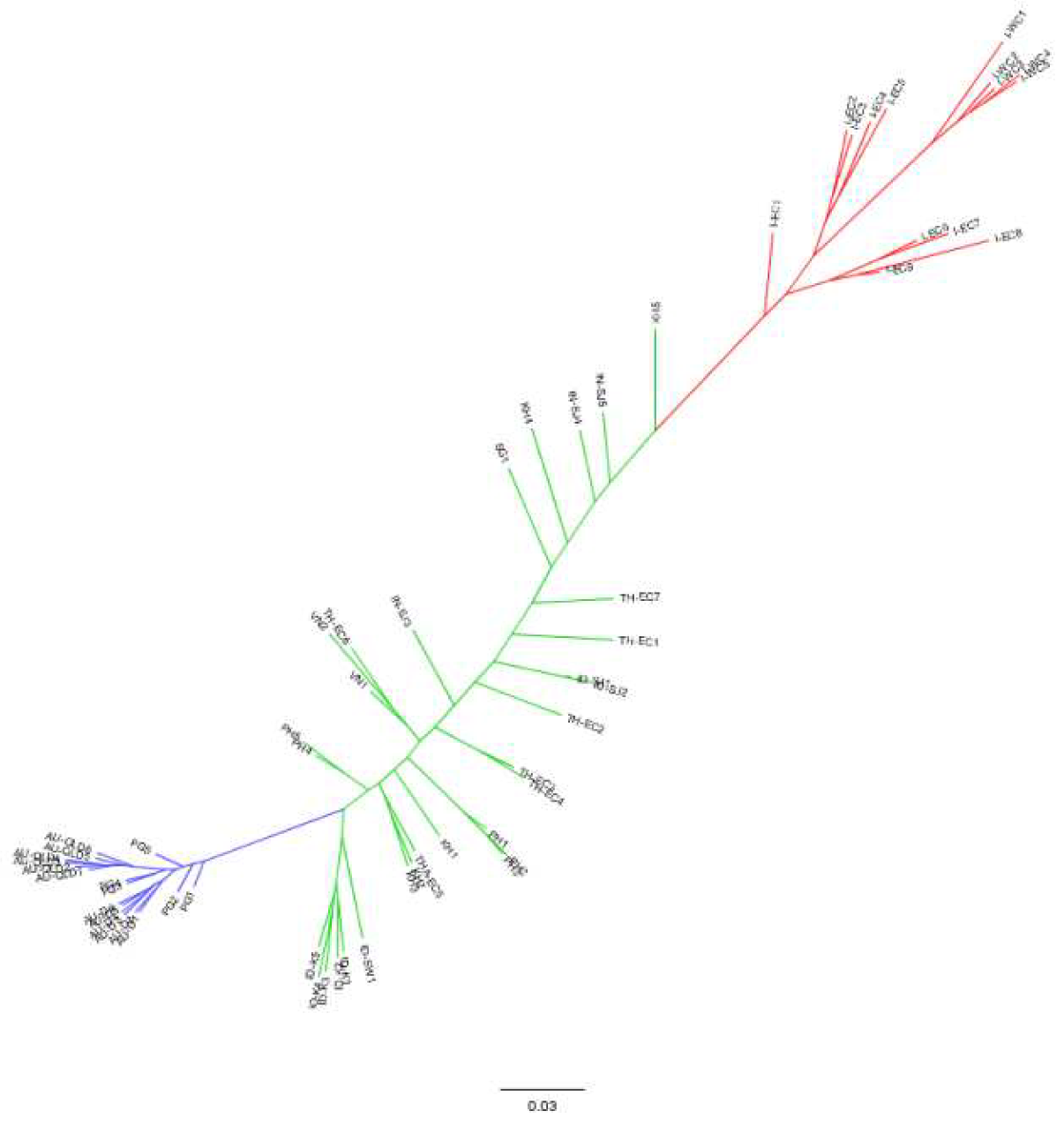

Supplement: S10 Fig — (TIF) [file pgen.1005954.s011.tif]

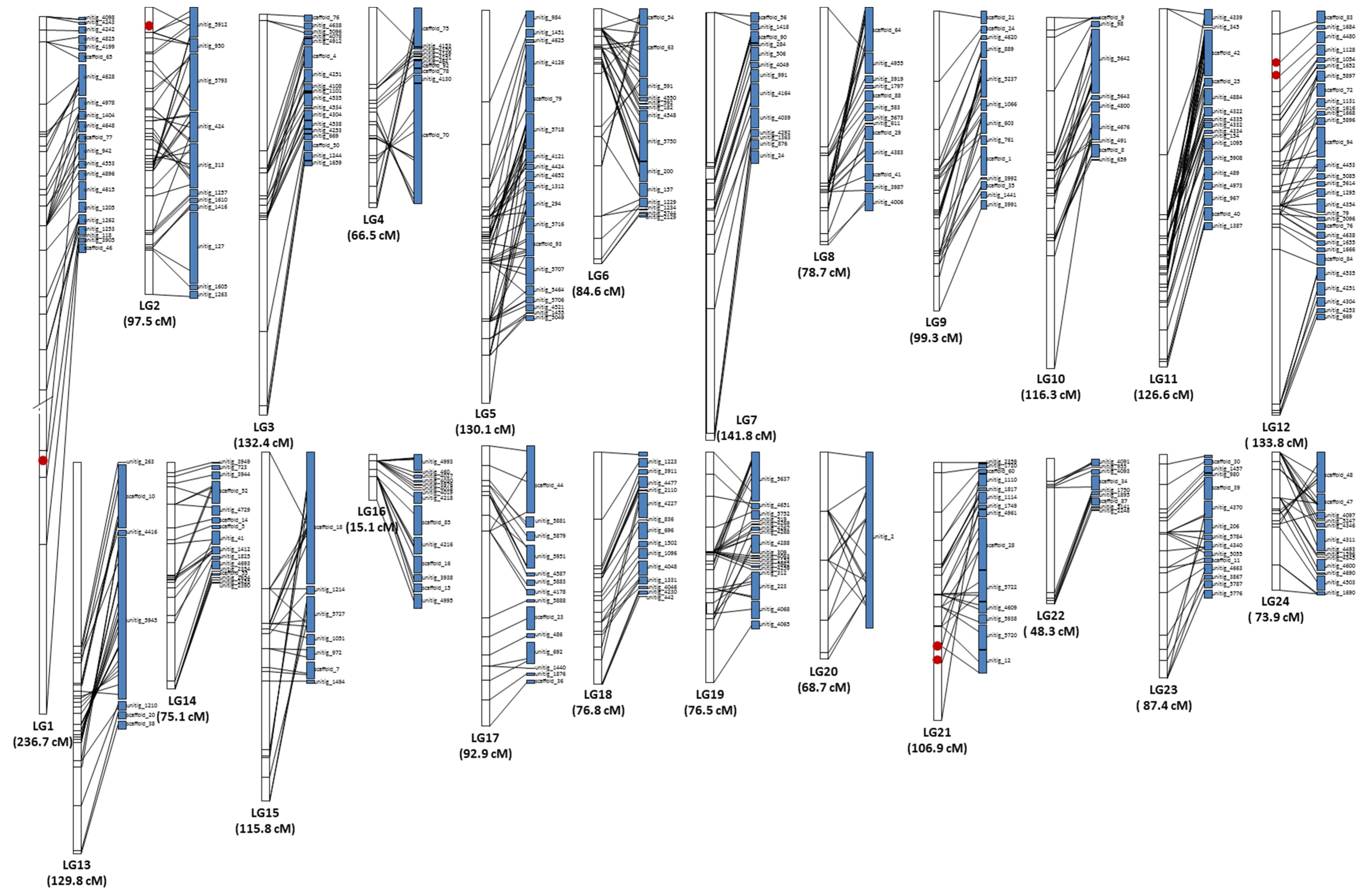

Supplement: S11 Fig — Regions indicated in red represent positions of contig/scaffold containing Lca_217 (peri-centromeric sequences). (TIF) [file pgen.1005954.s012.tif]

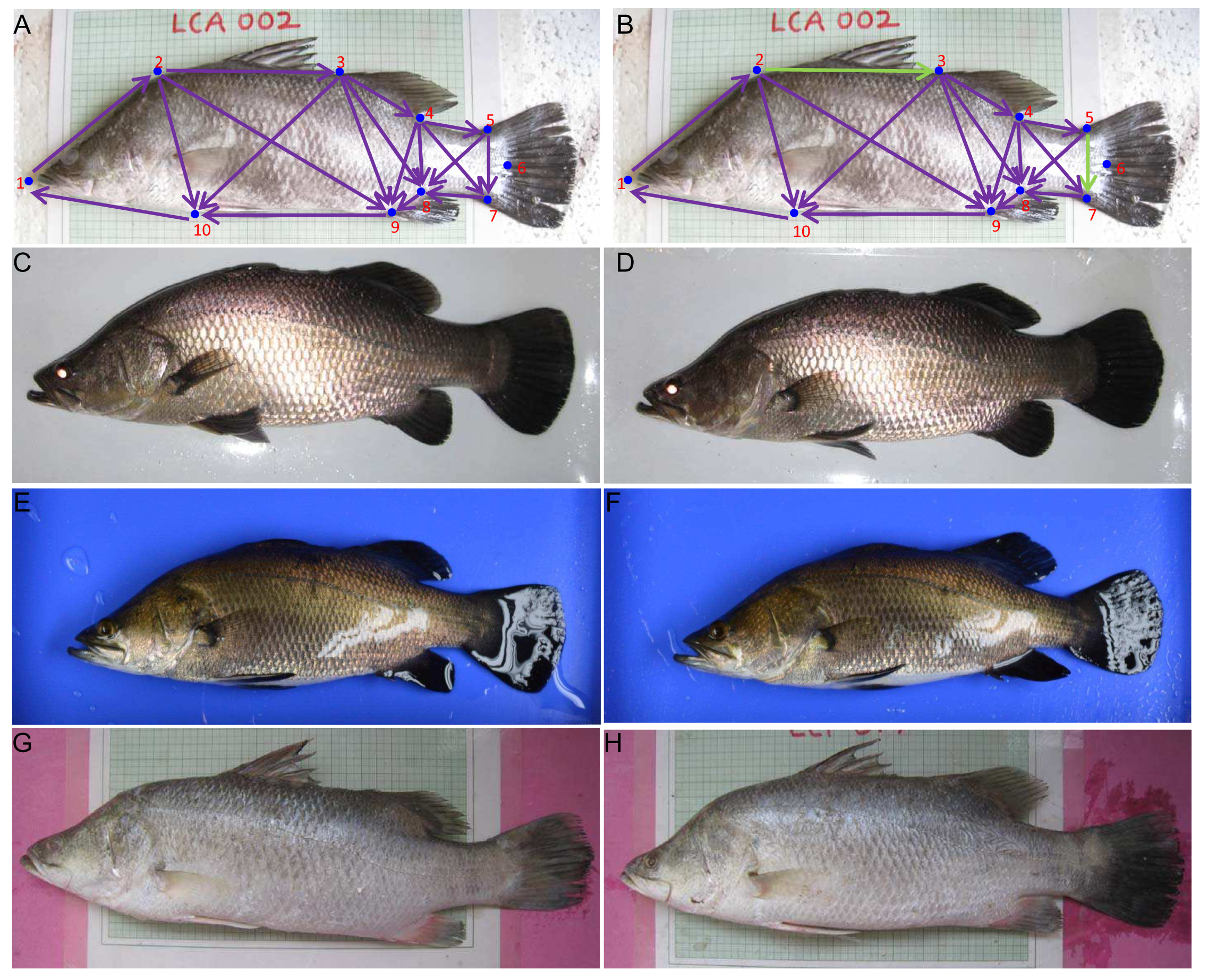

Supplement: S12 Fig — Purple and green lines represent truss measurements with blue circles indicating the landmark regions. The descriptions for the landmarks are, 1—tip of the snout, 2—point on dorsal surface of fish that is exactly perpendicular to the base of pectoral fin, 3—anterior base of dorsal fin, 4—posterior base of dorsal fin, 5—dorsal base of caudal fin, 6—base of central caudal fin rays, 7—ventral base of caudal fin, 8—posterior base of anal fin, 9—anterior base of anal fin and 10—base of pelvic fin (A & B). The landmark 6 is utilized only for generating the standard length of the fish. The remaining 9 landmarks were used to generate 18 inter-landmark truss measurements as indicated in panel A. The variables V18 and V3 (indicated with green colour in panel B) were observed to be more important for discriminating the three groups of fishes than other variables based on Wilks’ lambda criterion, coefficients of discriminant function and coefficients of structure matrix. Representative fishes from Australia-Papua New Guinea (C&D), SE Asia (E&F) and Indian region (G&H) are shown. (TIF) [file pgen.1005954.s013.tif]

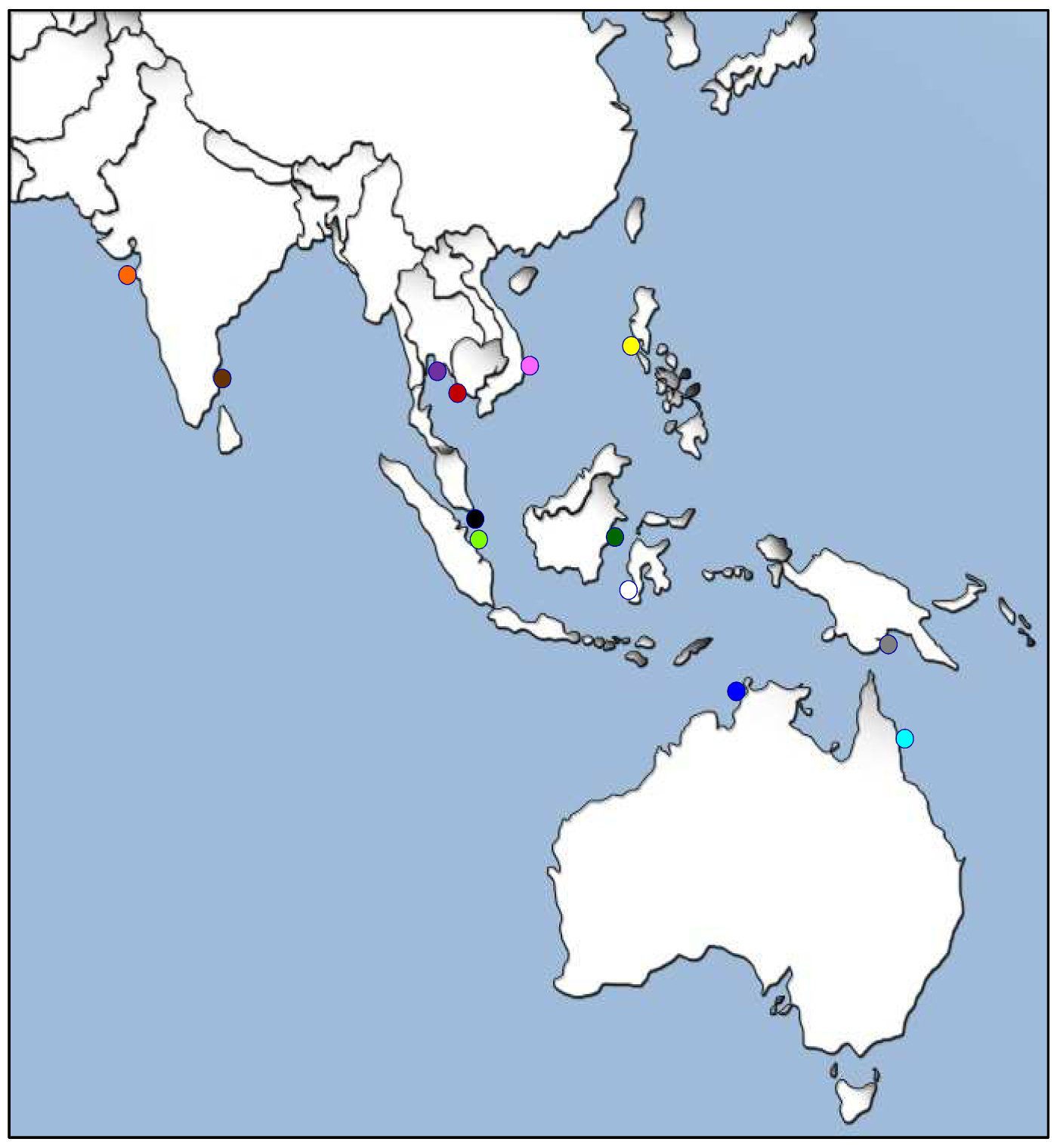

Supplement: S13 Fig — India-Western coast (orange), India-Eastern coast (brown), Cambodia (red), Thailand-Eastern Coast (purple), Vietnam (pink), Singapore (black), Philippines (yellow), Indonesia-South Jakarta (green), Indonesia-Kalimantan (dark green), Indonesia-Sulawesi (white), Papua New Guinea (grey), Australia-Darwin (blue) and Australia-Queensland (light blue). (TIF) [file pgen.1005954.s014.tif]

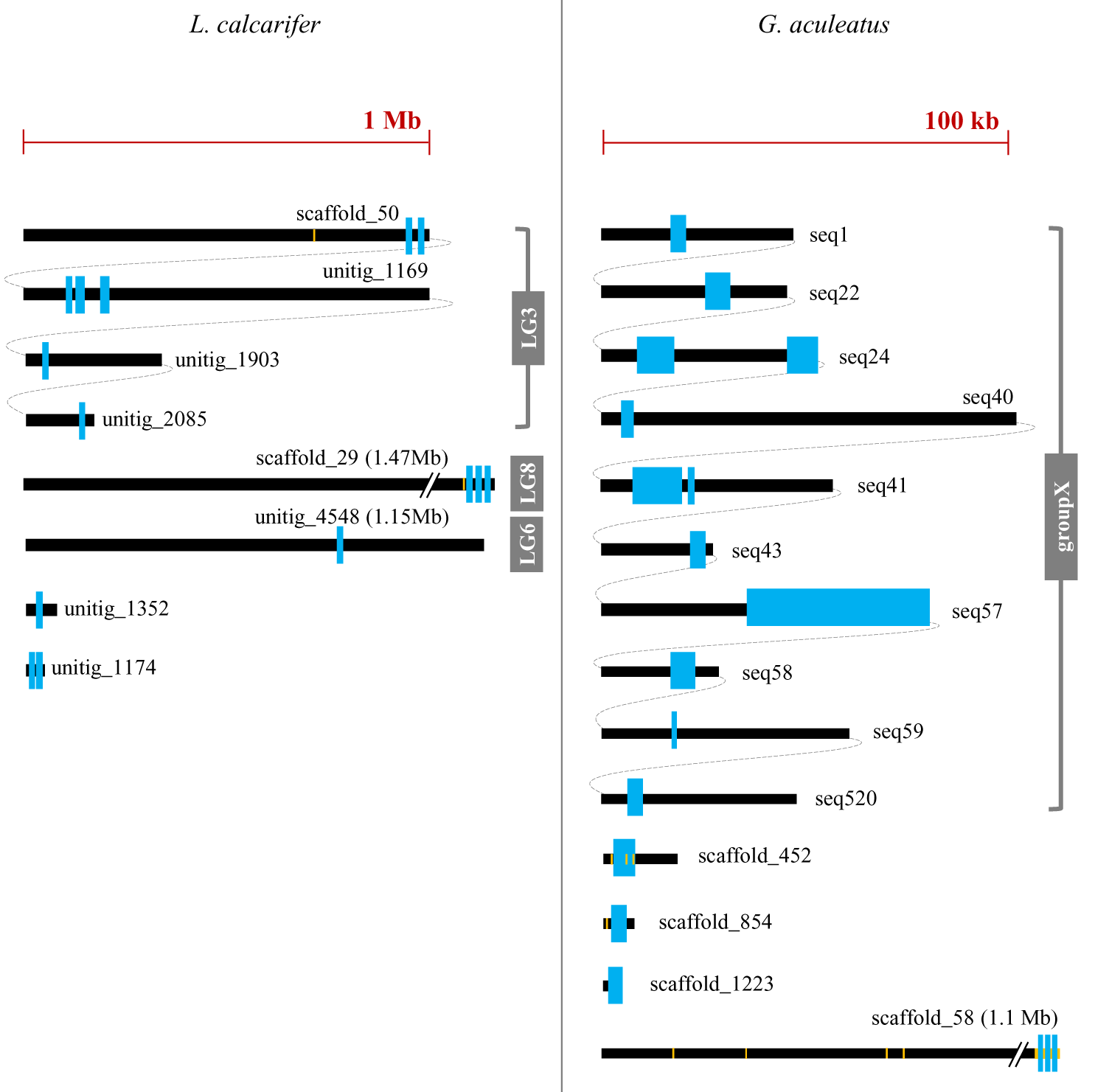

Supplement: S14 Fig — The L. calcarifer MHC-class I genes were found to be located on eight contigs/scaffolds, four of which were placed onto linkage group 3 (LG3). Four of these eight contigs/scaffolds were also >1Mb in length. The dashed connecting-lines indicate gaps introduced during sequence placement of contigs/scaffolds into linkage groups, while the yellow bars within the “scaffold_” sequences indicate Ns introduced during scaffolding. To allow for comparison at the level of contigs/scaffolds, the G. aculeatus chromosome groupX was split at the gapped regions (indicated by the dashed connecting-lines). The G. aculeatus MHC-class I genes were found to occupy 14 contigs/scaffolds, all except one being <113 kb in length. (TIF) [file pgen.1005954.s015.tif]
